# Canonical cytosolic iron-sulfur cluster assembly and non-canonical functions of DRE2 in *Arabidopsis*

**DOI:** 10.1371/journal.pgen.1008094

**Published:** 2019-04-29

**Authors:** Xiaokang Wang, Xudong Chen, Linhua Sun, Weiqiang Qian

**Affiliations:** 1 State Key Laboratory of Protein and Plant Gene Research, School of Life Sciences, and Peking-Tsinghua Center for Life Sciences, Peking University, Beijing, China; 2 Academy for Advanced Interdisciplinary Studies, and Peking-Tsinghua Center for Life Sciences, Peking University, Beijing, China; The University of North Carolina at Chapel Hill, UNITED STATES

## Abstract

As a component of the Cytosolic Iron-sulfur cluster Assembly (CIA) pathway, DRE2 is essential in organisms from yeast to mammals. However, the roles of DRE2 remain incompletely understood largely due to the lack of viable *dre2* mutants. In this study, we successfully created hypomorphic *dre2* mutants using the CRISPR/Cas9 technology. Like other CIA pathway mutants, the *dre2* mutants have accumulation of DNA lesions and show constitutive DNA damage response. In addition, the *dre2* mutants exhibit DNA hypermethylation at hundreds of loci. The mutant forms of DRE2 in the *dre2* mutants, which bear deletions in the linker region of DRE2, lost interaction with GRXS17 but have stronger interaction with NBP35, resulting in the CIA-related defects of *dre2*. Interestingly, we find that DRE2 is also involved in auxin response that may be independent of its CIA role. DRE2 localizes in both the cytoplasm and the nucleus and nuclear DRE2 associates with euchromatin. Furthermore, DRE2 directly associates with multiple auxin responsive genes and maintains their normal expression. Our study highlights the importance of the linker region of DRE2 in coordinating CIA-related protein interactions and identifies the canonical and non-canonical roles of DRE2 in maintaining genome stability, epigenomic patterns, and auxin response.

## Author summary

The Cytosolic Iron-sulfur cluster Assembly (CIA) pathway is essential for the maturation of Fe-S proteins localized in the cytosol and the nucleus. As an important component of the CIA pathway, DRE2 is essential from yeast to mammals. To study the CIA-related functions of DRE2 and further explore novel non-CIA roles of DRE2 in *Arabidopsis*, we for the first time created two homozygous *dre2* hypomorphic mutants using the CRISPR/Cas9 technology. The *dre2* mutants exhibit hallmark features of the CIA pathway mutants indicating CIA-dependent functions of DRE2 in *Arabidopsis*. Unexpectedly, we find that DRE2 participates in auxin response and nuclear DRE2 directly binds multiple auxin responsive genes and regulates their expression, suggesting that DRE2 plays CIA-independent roles. Our findings significantly expand our understanding of the biological functions of DRE2 in eukaryotes.

## Introduction

Iron-sulfur (Fe-S) proteins, which have Fe-S clusters mainly ligated to their cysteine residues, are ubiquitous in all three domains of organisms in nature: Bacteria, Archaea, and Eukarya. Depending on Fe-S clusters as cofactors, which play roles in electron transport, catalysis and regulation of gene expression, Fe-S proteins participate in diverse biological processes including respiration, photosynthesis, amino acid and purine metabolism, DNA replication and repair [1,2]. The maturation of Fe-S proteins involves synthesis of Fe-S clusters first and then assembly of Fe-S clusters on recipient proteins. Three pathways have evolved in eukaryotes for the maturation process, including the SUF (Sulfur mobilization) pathway in the plastids, the ISC (Iron-Sulfur Cluster assembly) pathway in the mitochondria and the CIA (Cytosolic Iron-sulfur cluster Assembly) pathway in the cytoplasm [3–6].

The maturation of cytoplasmic and nuclear Fe-S proteins is accomplished through a part of the ISC pathway and the CIA pathway. In yeast, the reactions carried out by the cysteine desulfurase complex Nfs1-Isd11 in the ISC pathway produce persulfide [7], which is transported from the mitochondria to the cytoplasm, in the form of glutathione trisulphide, by the ATP-binding cassette transporter Atm1 [8] and serves as the source of sulfur. Iron is provided via unknown mechanisms. In the cytoplasm, Fe-S clusters are assembled on the P-loop NTPases Cfd1–Nbp35 heterotetramer scaffold complex [9–11]. The newly synthesized iron-sulfur cluster is then transferred to recipient proteins with the assistance of the CIA targeting complex, which is composed of Cia1, Cia2 and Met18 [5,12–15]. Nar1 interacts with both Cfd1–Nbp35 scaffold complex and the CIA targeting complex and likely couples the two steps of Fe-S protein maturation [5,16]. Plants lack the homolog of yeast Cfd1 and NBP35 performs the scaffolding function in a homodimeric form. However, plant homologs of other yeast ISC and CIA proteins, including NFS1 [4], ISD11 [4], ATM3 (homolog of yeast Atm1) [17,18], CIA1 [4], AE7 (homolog of yeast Cia2) [19], MET18 [20–22] and NAR1 [23], are present and functional studies revealed that ATM3, AE7 and MET18 act in the CIA pathway as their yeast counterparts.

Dre2 (Derepressed for Ribosomal protein S14 Expression, also called CIAPIN1) was also identified as a component important for the CIA pathway in yeast [24]. Upstream of Nbp35, Dre2 forms a complex with the diflavin reductase Tah18. Electron transfer from NAPDH to Dre2 provides reducing power to Fe-S cluster assembly [25]. Dre2 itself contains a [2Fe-2S] cluster and a [4Fe-4S] cluster [24,26], which cannot be transferred to Fe-S proteins [25]. The assembly of Fe-S clusters on Dre2 does not rely on Tah18, Nbp35, Nar1 and Cia1 [25]. Studies in human cells revealed that the [2Fe-2S] clusters of anamorsin, human homolog of Dre2, can be transferred from a protein complex comprising human cytosolic monothiol glutaredoxin-3 (GRX3) and BOLA2 [27,28]. Plant homologs of yeast Tah18, Dre2 and human GRX3 are ATR3 [29], DRE2 and GRXS17 [30], respectively. DRE2 interacts with GRXS17 [30], ATR3 [29], and NBP35 [31]. However, CIA-related functional studies of DRE2 in plants remain undone. Intriguingly, it was found that epigenetic activation of maternal *FLOWERING WAGENINGEN* (*FWA*) in the central cell and endosperm, requires DRE2, but not other CIA proteins, and DRE2 also localizes in the nucleus [32], suggesting that DRE2 plays non-CIA roles.

DRE2-null mutants, like null mutants for other CIA proteins, except MET18, are embryonic lethal and no viable alleles of *DRE2* have previously been found. However, study of CIA-related functions of DRE2 and discovery of novel non-CIA roles of DRE2 demand viable alleles. In this study, we created hypomorphic *Arabidopsis* mutants for *DRE2* using the CRISPR/Cas9 system. Our genetic and biochemical evidence supports that the CIA-dependent role of DRE2 is important for the maintenance of genome and epigenome stability. Importantly, we find that DRE2 is involved in auxin response that may be independent of its CIA role. Our study reveals multiple CIA-dependent and -independent functions of DRE2 in plants.

## Results

### CRISPR/Cas9 generates viable mutant alleles of *DRE2*

**Fig 1:**
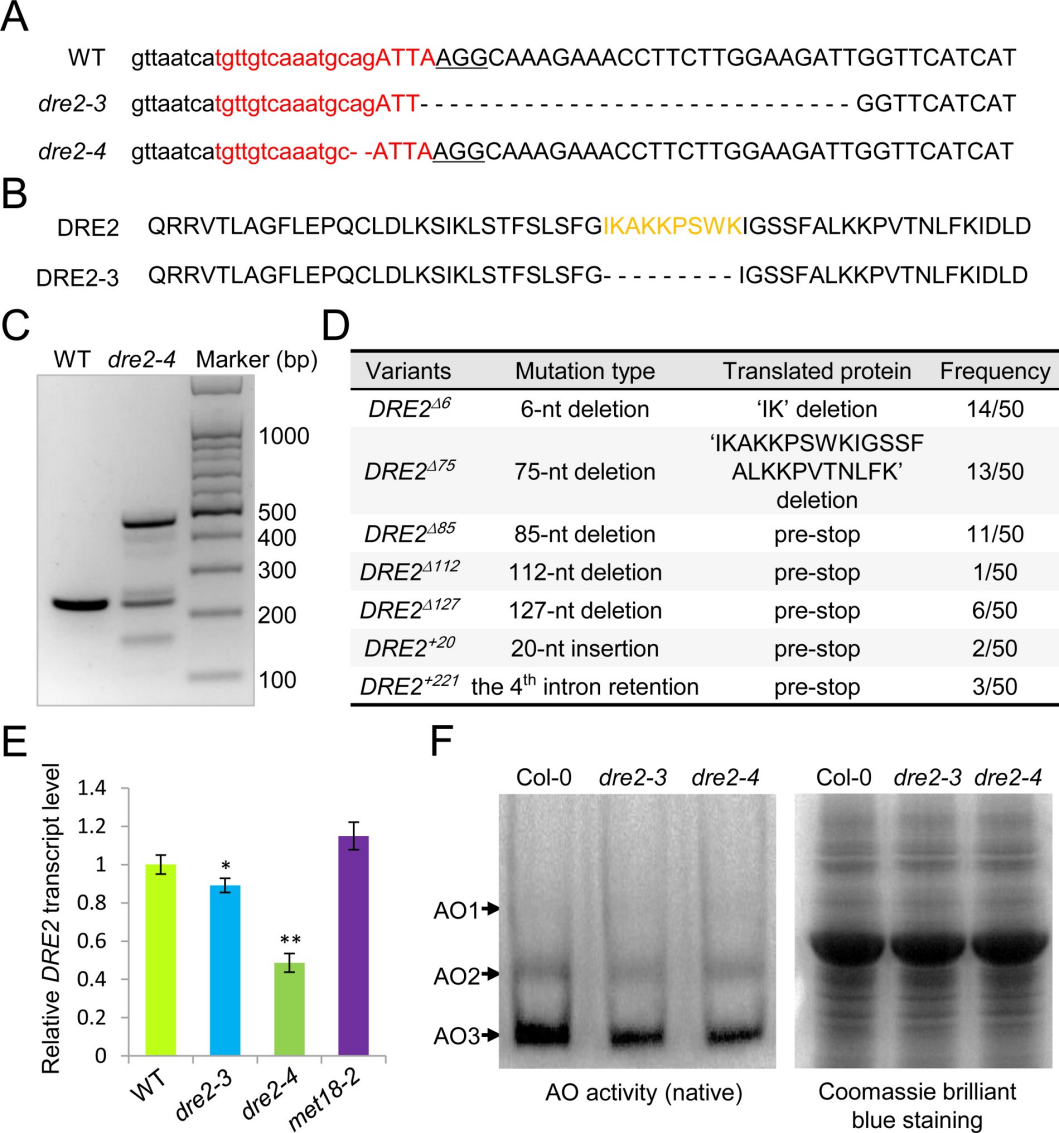
Generation of *dre2* hypomorphic mutants using the CRISPR/Cas9 system. (A) CRISPR/Cas9-induced deletions in *dre2-3* and *dre2-4*. The sgRNA-3 targeting sequence is highlighted in red and the PAM site is underlined. Exons are in upper case letters and introns in lower case. (B) The *dre2-3* mutation causes a deletion of nine amino acids. (C) The *dre2-4* mutation causes a splicing defect of *DRE2* pre-mRNA. RT-PCR shows that the splicing variants of *DRE2* mRNA in *dre2-4*. The positions of primers for RT-PCR are indicated by blue arrows in S1F Fig. (D) Summary of the splicing variants of *DRE2* mRNA in *dre2-4*. (E) Relative expression levels of *DRE2* in the indicated genotypes as determined by RT-qPCR. Data are presented as mean ± SD of four technical replicates. Asterisks indicate two-tailed Student’s t-test, *P < 0.05, **P < 0.01. Results from the second biological replicate is shown in S1D Fig. (F) In-gel activities of AO in Col-0 and *dre2* mutants. Equal amounts of protein (400 μg) extracted from seedlings were separated on nondenaturing PA gels and stained using synthetic aldehydes (1-naphthaldehyde) as substrates. Coomassie brilliant blue staining shows equal loading. The positions of the three bands, AO1, AO2, and AO3, are indicated with arrows.

Because complete loss of DRE2 functions leads to embryonic lethality [32], which precludes investigation of DRE2’s functions, we set out to create *dre2* hypomorphic mutants. For this purpose, we designed sgRNA1 to sgRNA3 targeting the first exon (corresponding to the N-terminal Methyltransferase-like domain [33]), the sixth exon (corresponding to the C-terminal CIAPIN1 domain [33]) and the junction of the fourth intron and exon (corresponding to the start of linker region), respectively (S1A and S1B Fig). Mutant lines carrying sgRNA1 or sgRNA2 developed chlorotic leaf spots or stripes reminiscent of cell death (S1C Fig). However, we were unable to obtain homozygous lines, indicating that these homozygous lines have lethal defects. Fortunately, we obtained two homozygous *dre2* mutant lines from the transformants expressing sgRNA3. The first line, named as *dre2-3*, harbors a 27-nucleotides deletion in the fourth exon (Fig 1A), which causes a deletion of the amino acids ‘IKAKKPSWK’ (Fig 1B). The second line, named as *dre2-4*, harbors a deletion of the 3′ splicing site ‘AG’ in the fourth intron (Fig 1A), which disrupts splicing of the *DRE2* mRNA (Fig 1C). We cloned *DRE2* cDNA from *dre2-4*. Seven splicing variants were identified from 50 clones we sent for sequencing. Two of them are deletion variants which deletes amino acids (*DRE2*^*Δ6*^ and *DRE2*^*Δ75*^). The rest of them all contain premature stop codons, which are resulted from either deletion (*DRE2*^*Δ85*^, *DRE2*^*Δ112*^ and *DRE2*^*Δ127*^), insertion (*DRE2*^*+20*^) or one intron-retention (*DRE2*^*+221*^) (Fig 1D; S1F and S1G Fig). No normal *DRE2* transcripts was identified from those clones, suggesting that the primary 3′ splicing site ‘AG’ is important for correct splicing of *DRE2* mRNA (S1F Fig). *DRE2* transcripts are slightly decreased in *dre2-3*, while reduced to about 40% in *dre2-4* (Fig 1E; S1D Fig). Like mutant lines carrying sgRNA1 or sgRNA2 (S1C Fig), *dre2-4* has chlorotic mosaics and wrinkled leaves (S1E Fig). To verify that the CIA-dependent function of DRE2 is at least partially compromised, we analyzed the activities of aldehyde oxidases (AO) [17], a representative Fe-S containing enzyme, in *dre2-3 and dre2-4*. In-gel AO activity staining revealed that the AO activities are indeed lower in *dre2-3* and *dre2-4* than that in Col-0 (Fig 1F). However, we did not find seed abortion in *dre2-3* and *dre2-4*. We further introduced the *pFWA*::*ΔFWA-GFP* reporter into *dre2-4*. The expression of *ΔFWA-GFP* in the central cell and endosperm was normal in *dre2-4* (S2 Fig), suggesting that *dre2-4* does not affect epigenetic activation of *FWA*. Thus, *dre2-3* and *dre2-4* may only lose some of DRE2’s functions.

### The viable mutants of *DRE2* have accumulation of DNA damage and constitutively activation of DNA damage response

**Fig 2:**
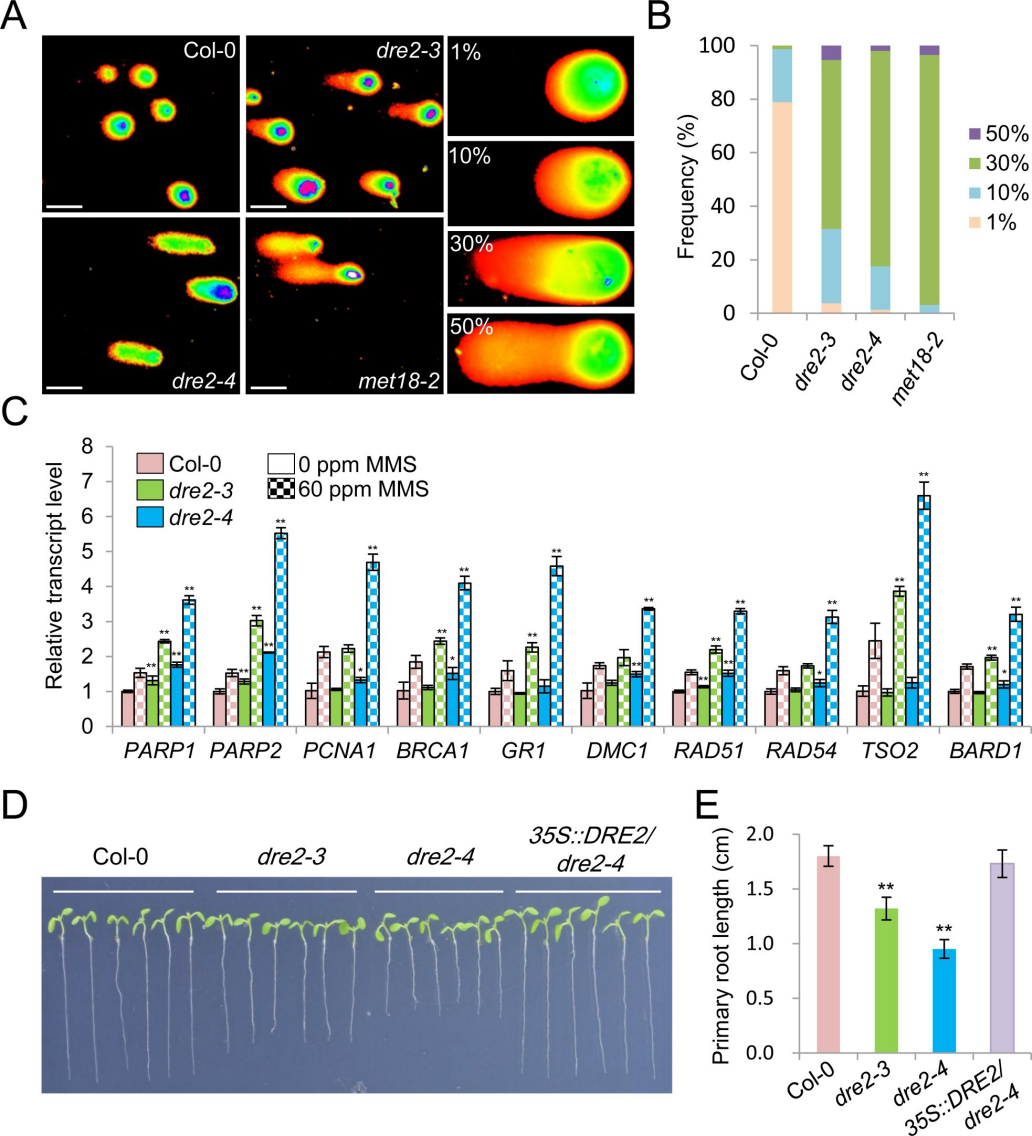
The *dre2* mutants have accumulation of DNA damage and constitutive activation of DNA damage response. (A) Typical comet images of nuclei from 21-day-old leaves of Col-0, *dre2-3*, *dre2-4* and *met18-2*. DNA damage was expressed as the ratio of the signal intensity of a comet tail versus that of the entire nucleus. (B) Frequency distribution of different grades of DNA damage in Col-0, *dre2-3*, *dre2-4* and *met18-2*. At least 200 comets were counted for each genotype. (C) Relative expression levels of the indicated genes in the indicated genotypes as determined by RT-qPCR. Data are presented as mean ± SD of four technical replicates. Asterisks indicate two-tailed Student’s t-test, *P < 0.05, **P < 0.01. Statistic tests are performed between Col-0 and the *dre2* mutants either with or without MMS treatment. Results from the second biological replicate are shown in S4 Fig. (D) Short root phenotype of the *dre2* mutants. Images of 7-day-old Col-0, *dre2-3*, *dre2-4* and *35S::DRE2*/*dre2-4* grown on vertical 1/2 MS plates were taken. (E) Statistic analysis of primary root lengths of 7-day-old Col-0, dre2-3, dre2-4 and 35S::DRE2/dre2-4 seedlings. Mean ± SD, n = 10; Asterisks indicate two-tailed Student’s t-test, *P < 0.05, **P < 0.01.

The CIA pathway mutants, including the *ae7*, *met18* and *grxs17* mutants, are characteristic of a compromise in DNA repair and accumulation of DNA damage [19,20,22,30]. To test whether the *dre2* mutants have the same defects, we conducted comet assays using the alkaline unwinding/neutral electrophoresis (A/N) protocol, which measures DNA SSBs as well as double strand breaks (DSBs) (Fig 2A). Based on relative signal intensities of the comet tail, we classified a broad spectrum of DNA damage into four levels: 1%, 10%, 30% and 50%. The majority of cells had 1% DNA damage and less than 2% cells had 30% and higher level of DNA damage in Col-0. However, the percentages of cells with 1% DNA damage dramatically decreased and the percentages of cells with 30% and 50% DNA damage increased to 65% or higher in *dre2-3*, *dre2-4* and *met18-2* (Fig 2A and 2B), suggesting accumulation of DNA damage in these mutants. In line with the comet assay results, *dre2-4* showed higher sensitivity to MMS, a DNA double strand break (DSB)-inducing chemical, than Col-0 (S3A Fig).

We further detected the expression levels of multiple genes involved in DNA Damage Response (DDR), including *PARP1*, *PARP2* [34], *PCNA* [35], *BRCA1*, *GR1*, *DMC1*, *RAD51*, *RAD54*, *TSO2* and *BARD1* [36] in Col-0, *dre2-3* and *dre2-4* without or with the application of MMS. We found that *PARP1*, *PARP2*, *PCNA*, *BRCA1*, *DMC1* and *RAD51* were activated in *dre2-3* and *dre2-4* even without MMS treatment, whereas the rest were activated only after MMS treatment (Fig 2C). Multiple cyclin genes, including *Cyclin-B1-1*, *Cyclin-B1-2*, *Cyclin-A1-1*, *Cyclin-A2-4* and *Cyclin-B2-4* were also upregulated in *dre2-3* and *dre2-4* (S3B Fig), suggesting that the accumulation of DNA damage and activation of DDR in these mutants arrested cell cycle progression. The short root phenotype is often associated with activation of DNA damage and cell cycle arrest. We also observed that *dre2-3* and *dre2-4* developed shorter roots than Col-0 (Fig 2D and 2E). It appeared that *dre2-4* is a stronger allele compared to *dre2-3*, as *dre2-4* had more DNA damage, more significant upregulation of DDR and cyclin genes and more severe short root phenotype (Fig 2). To exclude the possibility that these phenotypes are off-target effects arose from the CRISPR/Cas9 strategy, we introduced the *35S*::*DRE2* transgene into *dre2-4*. The upregulation of DDR genes and cyclin genes and the short root phenotype in *dre2-4* were all rescued by overexpression of wild type *DRE2* in *dre2-4* (Fig 2D and 2E; S4 Fig), suggesting that these phenotypes result from DRE2 dysfunction.

### The viable mutants of *DRE2* have DNA hypermethylation at hundreds of loci

**Fig 3:**
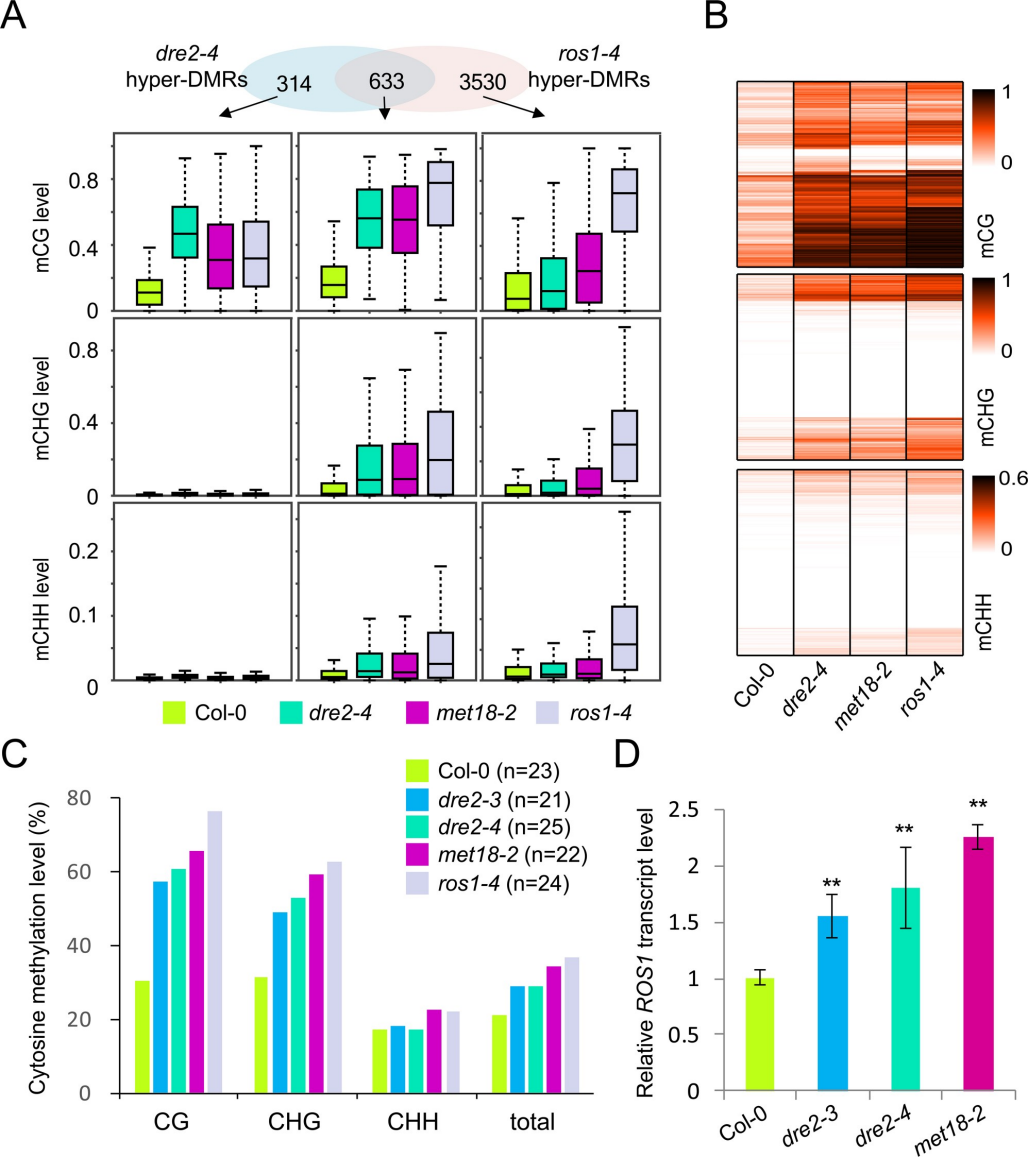
Dysfunction of *DRE2* causes genome-wide DNA hypermethylation. (A) Venn diagram showing the numbers of hyper-DMRs that either overlap between or are unique in *dre2-4* and *ros1-4*. Boxplots showing the distribution and the average CG, CHG, or CHH methylation levels calculated from hyper-DMRs in the respective subgroups. Dark horizontal line, median; edges of boxes, 25th (bottom) and 75th (top) percentiles; whiskers, minimum and maximum percentage of DNA methylation. (B) Heat-map showing the methylation levels of *dre2-4*-associated hyper-DMRs in Col-0, *dre2-4*, *met18-2* and *ros1-4*. The color key is presented at right. Light yellow indicates low methylation and black indicates high methylation. (C) Locus-specific bisulfite sequencing results showing the DNA methylation levels of the *ROS1* promoter in different genotypes. (D) Relative expression levels of *ROS1* in the indicated genotypes as determined by RT-qPCR. Asterisks indicate two-tailed Student’s t-test, *P < 0.05, **P < 0.01. Results from the second biological replicate are shown in S5D Fig.

The CIA pathway mutants, including the *ae7*, *met18* and *nbp35* mutants, have DNA hypermethylation at specific loci due to impaired activity of ROS1 [19,20,22,31], a DNA glycosylase which catalyzes the first step of active DNA demethylation [37]. To assess the effects of mutation of DRE2, an early-acting CIA factor, on DNA methylation patterns, we performed whole genome bisulfite sequencing on *dre2-4*. In total, we identified 1459 differentially methylated regions (DMRs) in *dre2-4*, including 951 hyper-DMRs and 508 hypo-DMRs (S5A Fig; S1 Dataset). Of the 951 hyper-DMRs, 633 (66.6%) are overlapping with those in *ros1-4* (Fig 3A; S5A Fig). Hyper-DMRs unique to *dre2-4* are mainly hyper-methylated in CG context (Fig 3A). The CG methylation levels of these *dre2-4* specific hyper-DMRs in *ros1-4* are also higher than those in Col-0, suggesting that hyper-methylated regions in *dre2-4* and *ros1-4* actually overlap to a larger extent (Fig 3A). Heat maps show that the methylation levels of *dre2-4* hyper-DMRs in *met18-2* and *ros1-4* are also higher than that in Col-0 in CG, CHG, and CHH contexts (Fig 3B). Analysis of the distribution of the *dre2-4* hyper-DMRs in different genomic regions (gene body region, intergenic region, TEs out of gene region and TEs overlapping with gene region) revealed that more than 60% of the hyper-DMRs are distributed in gene body regions in *dre2-4* (S5B Fig) much more than that of *ros1-4*. Collectively, these results suggest that mutation of the early-acting CIA factor DRE2 has a similar effect on overall DNA methylation patterns as mutation of the late-acting MET18. We further examined whether *ROS1* promoter, hypermethylation of which induces *ROS1* expression [38,39], is hypermethylated in *dre2-4*, as in *met18-2*. We found similar hypermethylation of *ROS1* promoter in *dre2-4* and *met18-2* (S5C Fig). Results of Locus-specific bisulfite sequencing confirmed this (Fig 3C). As a result of *ROS1* promoter hypermethylation, *ROS1* transcripts are upregulated in *dre2-3*, *dre2-4*, *met18-2* and *ros1-4* (Fig 3D). The upregulation of *ROS1* expression was also rescued by overexpression of wild type *DRE2* in *dre2-4* (S5D Fig). Despite upregulation of *ROS1* transcripts, the DNA hypermethylation phenotype of *dre2-4* exists and persists, suggesting impaired ROS1 activity.

### DRE2 mutations affect protein-protein interactions in the CIA pathway

**Fig 4:**
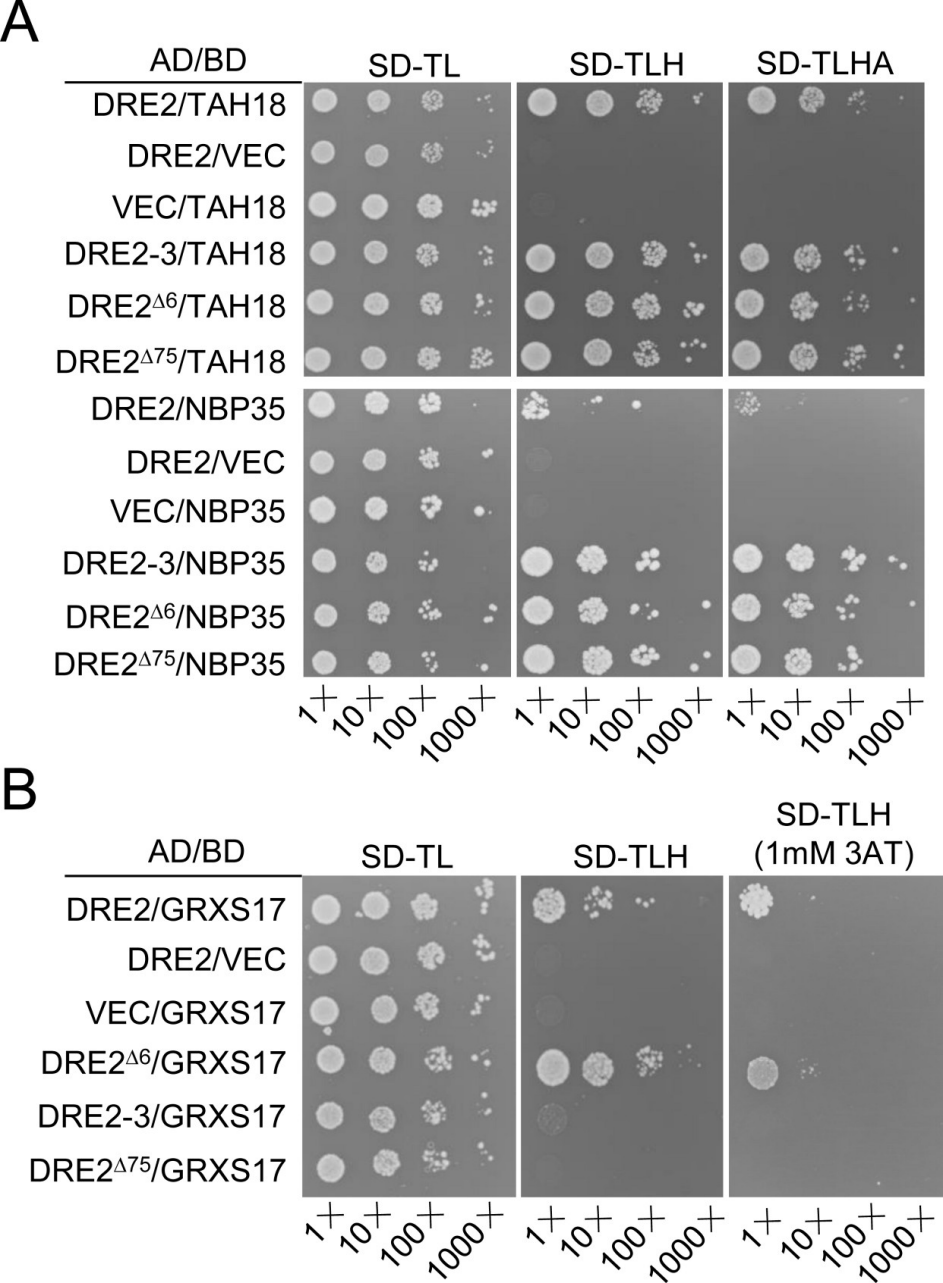
The linker region of DRE2 coordinates protein-protein interactions in the CIA pathway. (A-B) Interactions of DRE2, DRE2^*Δ*6^, DRE2-3 and DRE2^*Δ*75^ with TAH18, NBP35 and GRXS17 as determined by Y2H assays. Yeast cells harboring different fusion protein combinations (listed at the left) in pGBK-T7 (BD) and pGAD-T7 (AD) vectors were plated on medium lacking Leu, Trp (SD-TL), medium lacking Leu, Trp and His (SD-TLH) and medium lacking Leu, Trp, His and Ade (SD-TLHA) (A) or SD-TLH medium with 1 mM 3AT (B).

To investigate the underlying cause for defects in the *dre2-3* and *dre2-4* mutants, we carried out yeast two-hybrid (Y2H) assays to assess whether crucial protein-protein interactions are affected in *dre2-3* and *dre2-4*. As characterized above, a deletion mutant of DRE2, referred to as DRE2-3, is produced in *dre2-3*, whereas, two deletion mutants of DRE2, referred to as DRE2^Δ6^ and DRE2^Δ75^, are produced in *dre2-4* (Fig 1). Alignment of the deletion mutants with wild type DRE2 revealed that all of the deletions are located within the unstructured linker region that separate the N-terminal methyltransferase domain and the C-terminal CIAPIN1 domain [40] (S1B Fig). They start from Isoleucine139 (Ile139), but they end at different lysines (Ks) (S1G Fig). We first detected the interactions of these deletion mutants with TAH18 and NBP35. All of the deletion mutants interacted with TAH18, like wild type DRE2 (Fig 4A). However, they showed much stronger interaction with NBP35 compared with wild type DRE2 (Fig 4A). We next examined the interactions of these deletion mutants with GRXS17. While DRE2^Δ6^ interacted normally with GRXS17, DRE2-3 and DRE2^Δ75^ lost interaction with GRXS17 (Fig 4B). This is consistent with previous findings that the unstructured linker region of anamorsin in human cells reinforces its interaction with GRX3 [27]. Together, our results suggest that the linker region in DRE2 inhibits DRE2-NBP35 interaction but promotes DRE2-GRXS17 interaction.

### DRE2 is involved in auxin response

**Fig 5:**
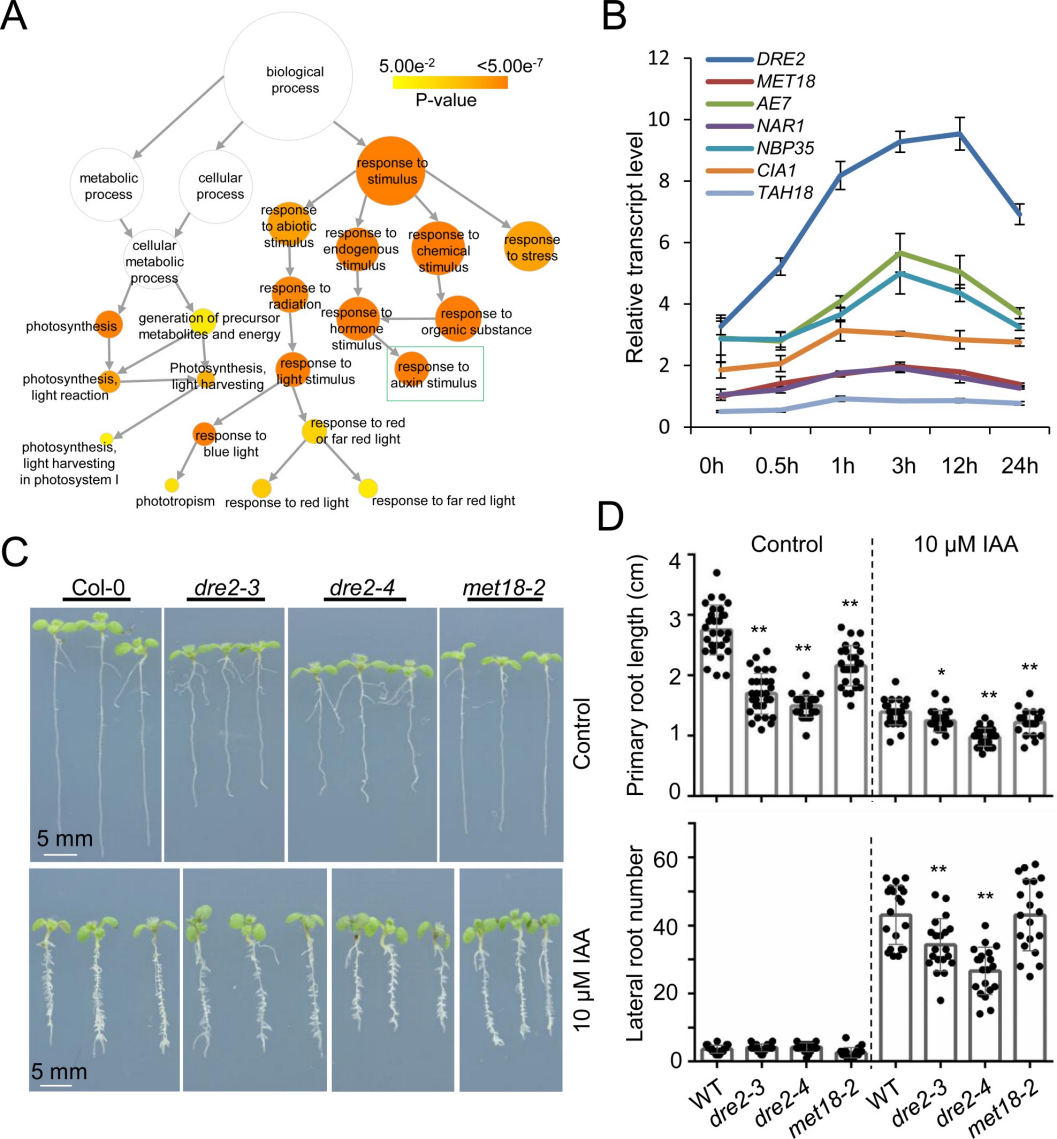
DRE2 is involved in auxin response. (A) Biological processes associated with downregulated genes in *dre2-4*. (B) Relative expression levels of CIA pathway genes after 10 μM IAA treatment. The expression level of *MET18* at 0 h was set as 1. Data are presented as mean ± SD of four technical replicates. (C) Primary and lateral root phenotypes of 9-day-old Col-0, *dre2-3*, *dre2-4* and *met18-2* without or with the application of 10 μM IAA. (D) Statistical analysis of primary root length (upper) and lateral root numbers (lower) of Col-0, *dre2-3*, *dre2-4* and *met18-2* seedlings. Mean ± SD, n ≥ 20; Asterisks indicate two-tailed Student’s t-test, *P < 0.05, **P < 0.01.

To uncover more biological functions of *DRE2*, we performed RNA-seq to identify the differentially expressed genes between 12-day-old Col-0 and *dre2-4* seedlings. Three biological replicates were performed and over 50 M high quality reads were obtained from the RNA library constructed from each sample. More than 90% of the reads could be uniquely mapped to the Tair10 *Arabidopsis* genome. In total, we identified 1593 genes that were significantly upregulated and 1111 genes that were significantly downregulated (fold-change>2, q<0.01) in *dre2-4* as compared to Col-0 (S2 Dataset). Gene Ontology (GO) analysis of the upregulated genes revealed 77 enriched biological processes (FDR<0.05), including ‘response to ionizing radiation’, ‘response to gamma radiation’, ‘cell cycle’ and ‘DNA replication’ (S6A and S6C Fig). This is in accordance with our results that mutations in *DRE2* cause accumulation of DNA damage and constitutive activation of DDR (Fig 2). GO analysis of the downregulated genes revealed 22 enriched biological processes (FDR<0.05), among which ‘response to auxin stimulus’ is enriched (Fig 5A; S6B and S6D Fig).

To confirm that DRE2 is involved in auxin responses, we first performed RT-qPCR to detect the expression levels of *DRE2* and other CIA pathway components after treatment with IAA, the natural auxin. While the expression of other CIA pathway proteins were mildly induced, the expression of *DRE2* was greatly induced by IAA, especially at early stage after IAA treatment (Fig 5B). Secondly, we tested whether the growth of the primary root, which is inhibited by auxin, is affected in the *dre2* mutants. The effect of IAA treatment on primary root length was much weaker in the *dre2* mutant compared to that in wild type and *met18-2* (Fig 5C and 5D; S7 Fig), indicating that the *dre2* mutations confer auxin insensitivity. Third, we tested whether the increase of lateral root formation, that is promoted by auxin [41,42], is affected in *dre2-3*, *dre2-4* and *met18-2*. There is no significant difference of the lateral root numbers between Col-0 and the three mutants without the application of IAA (Fig 5C and 5D). IAA treatment greatly increased the lateral root numbers in Col-0 and the three mutants. The increase of lateral root number in *met18-2* is comparable to that in Col-0. However, the increase of lateral root numbers in *dre2-3* and *dre2-4* mutants was significantly less than that in Col-0 (Fig 5C and 5D). Together, our results suggest that DRE2 participates in auxin response. This could be a CIA-independent function of DRE2.

### DRE2 localizes in both the cytoplasm and the nucleus and nuclear DRE2 directly binds several auxin responsive genes

**Fig 6:**
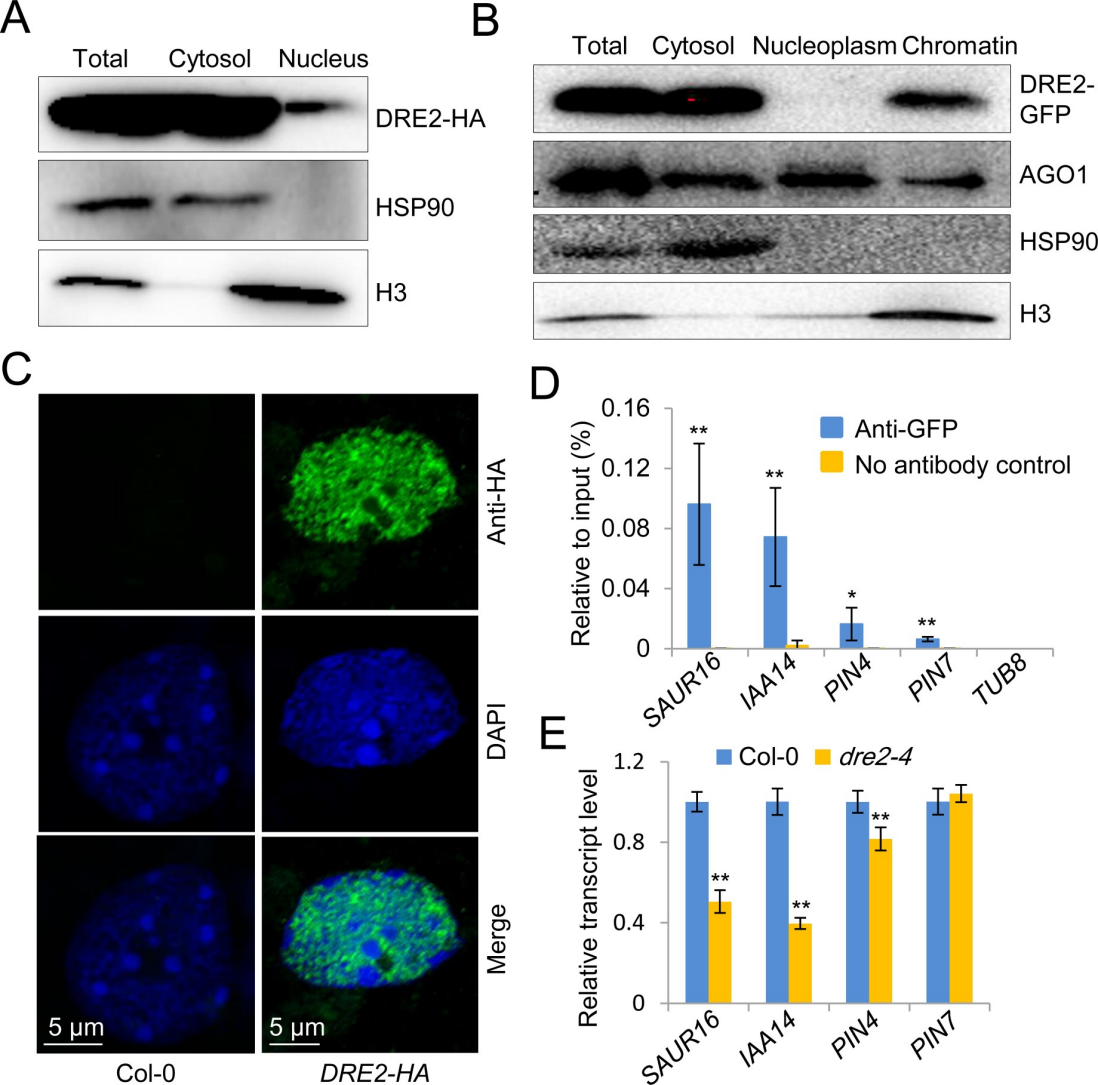
DRE2 associates with chromatin and some auxin responsive genes. (A-B) Detection of DRE2 in cytoplasmic, nucleoplasmic and chromatin-associated fractions by Western blot. DRE2-HA fusion protein was detected using an anti-HA antibody. DRE2-GFP fusion protein was detected using an anti-GFP antibody. HSP90 and histone H3 were detected as markers for cytoplasmic proteins and chromatin proteins, respectively. AGO1 was detected as markers for proteins present in all the fractions. (C) Immunostaining results showing the nuclear distribution of DRE2. DRE2-HA in the *pDRE2::DRE2-HA* transgenic plants was stained with an anti-HA antibody and an Alexa Fluor 488-conjugated secondary antibody (green). DNA was stained with DAPI (blue). (D) ChIP-qPCR results showing the enrichment of DRE2-GFP at four auxin responsive genes. (E) Relative expression levels of the indicated genes in the indicated genotypes as determined by RT-qPCR. Data are presented as mean ± SD of four technical replicates. Asterisks indicate two-tailed Student’s t-test, *P < 0.05, **P < 0.01.

Previous studies showed that DRE2 mainly localizes in the cytoplasm, like other CIA pathway proteins, but weak DRE2-GFP signal can be detected in the nucleus [32]. To ascertain the subcellular localization of DRE2, we generated *pDRE2*::*DRE2-GFP* transgenic plants in Col-0 background. Consistently, we found that DRE2-GFP mainly localized in the cytoplasm in the differentiation zone of root (S8A Fig). Strong nuclear DRE2-GFP signal could be detected after Leptomycin B (an exportin inhibitor) treatment (S8A Fig), suggesting that DRE2 shuttles between the nucleus and the cytoplasm. In the meristematic zone of root, DRE2-GFP appeared to be more abundant in the nucleoplasm than in the cytoplasm (S8B Fig). Unexpectedly, strong DRE2-GFP signal could be detected in the nucleolar cavity (S8B Fig). We further performed subcellular fractionation experiments with HSP90 and Histone H3 as cytoplasmic and nuclear protein markers, respectively. We found that DRE2 was present in both the cytoplasm and the nucleus (Fig 6A; S9 Fig). Importantly, when we separated the nuclear fraction into nucleoplasm and chromatin-bound fractions, we found that DRE2 was associated with chromatin (Fig 6B; S9 Fig). To further visualize the nuclear localization of DRE2, we immunostained nuclei isolated from Col-0 and the *DRE2-HA* transgenic plants. The DRE2-HA signal did not overlap with compacted heterochromatin regions that are intensely stained by DAPI, but overlapped with open euchromatin regions that are weakly stained by DAPI (Fig 6C), suggesting that DRE2 is specifically associated with euchromatin. To test whether DRE2 can directly bind auxin responsive genes, we performed chromatin immunoprecipitation (ChIP)-qPCR using *pDRE2*::*DRE2-GFP* transgenic plants. Our results revealed that DRE2 were enriched at some auxin responsive genes, including *SAUR16*, *IAA14*, *PIN4* and *PIN7* (Fig 6D). In *dre2-4* mutants, the transcript levels of *SAUR16*, *IAA14* and *PIN4* were reduced, while *PIN7* remained unaltered (Fig 6E). These results suggest that DRE2 binding is important for the expression of auxin responsive genes.

## Discussion

In this study, we created hypomorphic *dre2* mutant by CRISPR/Cas9 technology, and systematically explored the function of DRE2 using a combination of genetic and biochemical approaches. We demonstrate that DRE2 fulfills canonical CIA-related roles. It also participates in auxin response, which may be CIA-independent.

### CRISPR/Cas9 technology creates hypomorphic *dre2* mutants

For most of the essential genes, no homozygous cell lines or mutants can be obtained. This impedes the functional studies of these genes. RNAi and CRISPR/Cas9 are two technologies for gene silencing, with the former knock down genes and the latter knock out genes or introduce deletions/mutations. Because RNAi only reduces the expression of genes, it is a better choice for investigating the functions of essential genes. However, RNAi-mediated reduction of gene expression sometimes is not sufficient to result in desired molecular and/or phenotypic changes. For instance, the RNAi line for *NBP35* have unaltered activities of Fe-S enzymes in leaves [31]. Another drawback of RNAi is that it often has off-target effects and may produce false positive results [43]. CRISPR/Cas9 is effective for editing of specific genomic regions and has less off-target effects. Thus, it could be ideal for creation of hypomorphic mutants for essential genes. Usually, sgRNAs targeting exons are designed to introduce insertion or deletions mutations in protein-coding regions of genes. Different from the popular strategy, we designed a sgRNA targeting an intron-exon junction region of *DRE2* to create mis-splicing mutants for *DRE2*. We successfully created mis-splicing mutants for *DRE2* and obtained viable homozygous lines. The mutant lines were found to exhibit hallmark features of the CIA pathway mutants. Moreover, we found that DRE2 participates in auxin response independently of its CIA role using these mutant lines. Our results suggest that generating viable mutant lines using CRISPR/Cas9 technology is a good strategy for studying essential genes.

### The *dre2* mutants exhibit hallmark features of the CIA pathway mutants

To clearly identify DRE2 as a CIA factor, we measured the activities of AO in *dre2-3 and dre2-4*. Like the *atm3* mutant [17], *dre2-3 and dre2-4* have low AO activities, suggesting that the CIA function of DRE2 is conserved in plants. Like the *ae7*, *met18*, *atm3* and *grxs17* mutants, the *dre2* mutants have accumulation of DNA damage and activation of DDR. In eukaryotes, Fe-S clusters have been found to be assembled on DNA replication/repair proteins, including Pol α, δ, ε, and ζ, which are DNA polymerases for DNA replication and repair [44], PriL, which is a subunit of eukaryotic primase for DNA replication and telomere maintenance [45,46], XPD and FancJ, which are DNA helicases involved in nucleotide excision repair [47], RTEL1, which is a DNA helicase involved in homologous recombination [48]. The accumulation of DNA damage and activation of DDR in the *ae7* [19], *met18* [15], *atm3* [19] and *grxs17* mutants have been proposed to be caused by defective assembly of Fe-S clusters on plant counterparts of these proteins. It is possible that the DNA damage phenotype of the *dre2* mutants is also a result of defective assembly of Fe-S clusters on DNA replication/repair proteins. Previous findings indicate that different Fe-S proteins are differentially affected by certain CIA mutation, future investigations are needed to determine which DNA replication/repair proteins are preferentially affected by DRE2.

The *ae7*, *nbp35* and *met18* mutants have low activity of ROS1 and whole genome bisulfite sequencing analysis revealed DNA hypermethylation at hundreds of loci in *met18* [19,20,22,31]. The *dre2* mutants also exhibit DNA hypermethylation and the pattern of DNA hypermethylation in *dre2-4* resembles that in *met18-2* (Fig 3B). Our results suggest that the action of DRE2, an early acting CIA factor, is also required for full enzymatic activity of ROS1. *ROS1* transcript levels did not decrease in the *dre2* mutants, excluding the possibility that decreased expression of *ROS1* caused DNA hypermethylation. AE7 and MET18 interact with ROS1 and the physical interactions may mediate the transfer of Fe-S cluster to ROS1 and contribute to ROS1 activity [19,20,22,31]. As DRE2 is not a component of the CIA targeting complex, it is less likely that DRE2 directly interacts with ROS1. Impairment of Fe-S assembly on ROS1 is less likely a result of disrupted DRE2-ROS1 interaction. As electron transfer from DRE2 provides reducing power to Fe-S cluster assembly, impairment of Fe-S assembly on ROS1 could be due to inadequate reducing power.

### The unstructured linker of DRE2 coordinates protein-protein interaction for proper CIA function of DRE2

The DRE2 protein contains a methyltransferase-like domain at the N-terminal, a CIAPIN1 domain at the C-terminal, and an unstructured linker in the middle (S1B Fig). In human cells, the unstructured linker is the only region of anamorsin that tightly interacts with Ndor1 (human homolog of Tah18) [49] and can stabilize the interaction with GRX3 [27,28]. The *dre2-3* and *dre2-4* mutations, we created by CRISPR/Cas9, lead to amino acid deletions in the unstructured liker region. Consistent with studies in human cells, our Y2H results revealed that the linker is required for DRE2’s interaction with GRXS17. However, in contrast to promoting DRE2-GRXS17 interaction, the linker region was found to inhibit DRE2’s interaction with NBP35. The opposing functions of the linker region on DRE2-GRXS17 interaction and DRE2-NBP35 interaction lead us to propose that before mature, DRE2 exposes its linker region to facilitate DRE2-GRXS17 interaction and Fe-S cluster transfer from GRXS17 to DRE2. Binding of Fe-S cluster and maturation of DRE2 may change the conformation of DRE2 and hide the linker region, thus disrupting DRE2-GRXS17 interaction to facilitate DRE2-NBP35 interaction and Fe-S cluster assembly on cytoplasmic or nuclear Fe-S proteins. Because the GRX3-anamorsin interaction promotes [2Fe-2S] cluster transfer [27], loss of DRE2-GRXS17 interaction before Fe-S transfer to DRE2 in *dre2-4* may lead to reduced Fe-S binding on DRE2, eventually leading to the CIA-related defects in *dre2-3* and *dre2-4*. The CIA-related defects in *dre2-3* and *dre2-4* may also arise from stronger interaction between DRE2 and NBP35.

### Novel functions of DRE2

The CIA function of DRE2 is considered to be executed by cytoplasmic DRE2. Previous studies found that DRE2 also localizes in the nucleus [32]. In this study, we confirmed the nuclear localization of DRE2 and found that it can associate with chromatin (Fig 6A and 6B). Further immunostaining results indicate that DRE2 is associated with euchromatin (Fig 6C). The nuclear localization, especially the euchromatin-association, of DRE2 suggests that DRE2 plays non-CIA roles. Previous studies revealed one of the non-CIA roles that DRE2 plays is activation of the imprinting gene *FWA* in the central cell and endosperm [32]. We found that *dre2* mutant lines created by sgRNA1 or sgRNA2 and *dre2-4* show chlorotic leaf spots or stripes reminiscent of cell death. This phenotype was detected only in the *dre2* mutants, but not in other CIA pathway mutants, suggesting that DRE2 may regulate signaling pathways related to cell death independently of its CIA role. Through RNA-seq analysis, we found that auxin response was disrupted in *dre2* mutants. Because only *DRE2* was greatly induced by auxin treatment and only *dre2* mutants, but not Col-0 and *met18-2*, have alleviated inhibition of primary root growth and less increase of lateral root number, we propose that the involvement of DRE2 in auxin response might be independent of its CIA role. Our ChIP-qPCR results revealed that DRE2 directly binds multiple auxin responsive genes and DRE2 binding is required for their optimal expression. Thus, it is very likely that DRE2 participates in auxin response by controlling the expression of auxin responsive genes, although we could not exclude the possibility that the CIA-dependent function of DRE2 indirectly (via a FeS protein) contributes to the auxin response. Interestingly, the knockout line and RNAi line for *GRXS17* also display reduced auxin sensitivity, albeit only at high temperature. Specifically, the inhibition of primary root length by auxin is alleviated in the knockout line and RNAi line for *GRXS17*. This was attributed to elevated levels of reactive oxygen species in these lines [50]. Our finding that DRE2, a downstream Fe-S transfer target of GRXS17, participates in auxin response suggesting that malfunction of DRE2 could be a cause of altered auxin response in these lines. Together, our findings define the nuclear localization of DRE2, its association with chromatin and its modulation of auxin responsive gene expression. It will be interesting to search for nuclear DRE2-interacting proteins and other nuclear DRE2 functions in the future.

## Materials and methods

### Plant materials and growth conditions

All *Arabidopsis* materials used in this study are in Columbia background. The *met18-2* (SALK_147068) mutant has been described previously [19,22]. The *dre2-3* and *dre2-4* mutants were generated by an egg cell-specific promoter-controlled CRISPR/Cas9 system [51] using *sgRNA-3* (S1 Table). For horizontally-grown seedlings, surface-sterilized seeds were germinated on 1/2 Murashige-Skoog (MS) medium (7‰ Agar and 1% Suc) at 22°C with 16 h of light and 8 h of darkness. For vertically-grown seedlings, surface-sterilized seeds were germinated on 1/2 MS medium (12‰ Agar and 1% Suc) also at 22°C with 16 h of light and 8 h of darkness. The seedlings were harvested for experiments or transplanted into soil and grown at 22°C with the same photoperiod.

To generate the *pDRE2*::*DRE2-GFP* and *pDRE2*::*DRE2-HA* transgenic plants, *DRE2* genomic DNA fragment with its native promoter was amplified from Col-0 genomic DNA by PCR and cloned into the *pCAMBIA1300* or *pCAMBIA1305* vectors for plant transformation. For complementation of *dre2-4*, *DRE2* coding sequence (CDS) was amplified from Col-0 cDNA by PCR and cloned into the *pCAM2300-35S-Ocs* vector for plant transformation. *Agrobacterium tumefaciens* strain GV3101 carrying different constructs were used to transform the wild type or mutant plants via the standard floral dipping method [52]. Primary transgenic plants were selected on 1/2 MS plates containing 25 mg/L hygromycin (*pCAMBIA1300* and *pCAMBIA1305*) or 50 mg/L kanamycin (*pCAM2300-35S-Ocs*). Homozygous transgenic lines were used for further experiments. The primers used for PCR are listed in S1 Table.

### Enzyme extraction and AO activity staining

Enzyme extraction and AO activity staining were performed as previously described [53–55]. Twelve-day-old seedlings were ground into fine powder and 100 mg of powder were suspended in 400 μL of extraction buffer (50 mM Tris-HCl (pH 7.5), 1 mM EDTA, 1 mM sodium molybdate, 10 μM flavin adenine dinucleotide, 2 mM DTT, protease inhibitor (one tablet/100 mL) and Polyclar AT (0.2 g/g fresh weight)). After centrifugation at 15,000 rpm for 20 min, the supernatant was concentrated by filtration using Amicon Ultra-0.5 Centrifugal Filters (3K) to achieve a final volume of 50 to 100 μL. The concentrated samples were used as crude enzyme preparations for native PAGE (6%). After electrophoresis, the gel was immersed in 0.1 M potassium phosphate (pH 7.4) for 5 min, and then the activity of AO was determined by developing in a reaction mixture containing 0.1 M potassium phosphate buffer (pH 7.4), 1 mM 1-naphthaldehyde, 0.1 mM phenazine methosulfate, and 0.4 mM MTT at room temperature (about 25°C) for 1 h in the dark.

### Real-time RT-PCR

Total RNA was extracted from 12-day-old seedlings grown on 1/2 MS medium using the TRIzol reagent (Invitrogen, 15596026). About 2 μg of total RNA was used for first-strand cDNA synthesis with the 5X All-In-One RT MasterMix (abm, G485) following the manufacturer’s instructions. The cDNA reaction mixture was then diluted 10 times, and 1 μL was used as a template in a 20 μL PCR reaction with PowerUp SYBR Green Master Mix (Applied Biosystems). The RNA transcript levels were determined by quantitative RT-PCR. *TUB8* was used as an internal control. The primers used for PCR are listed in Table S1.

### Comet assay

Comet assay was performed by using the Trevigen comet assay kit (Trevigen, 4250-050-K) following the manufacturer’s instruction. Briefly, nuclei extracted from 22-day-old rosette leaves at 1 x 10^5^/mL were combined with molten LMAgarose at a ratio of 1:10 (v/v) at 37°C. Samples (50 μL) were immediately pipetted onto CometSlide. The slides were placed flat at 4°C in the dark for 30 min and then immersed in prechilled Lysis Solution (Trevigen, 4250-050-01,) at 4°C for 60 min. After lysis, the slides were immersed in freshly prepared Alkaline Unwinding Solution, pH >13 (200 mM NaOH, 1 mM EDTA) for 20–60 min at room temperature in the dark. Electrophoresis was in Alkaline Electrophoresis Solution, pH >13 (200 mM NaOH, 1 mM EDTA) at 21 volts for 30 min at 4°C. After washing in dH_2_O and 70% ethanol, samples were dried at ≤ 45°C for 10–15 min. DNA was stained by diluted SYBR Green I (Trevigen, 4250-050-05,) in refrigerator for 5 min. The slides were dried completely at room temperature in the dark and then viewed by Olympus BX53 fluorescence microscope. The Comet Score software (http://www.autocomet.com) was used to evaluate the levels of DNA damage.

### Whole-genome bisulfite sequencing and data analysis

About 3 μg genomic DNA was extracted from 14-day-old seedlings using Hi-DNAsecure Plant Kit (TIANGEN, DP350-03) and then sent for bisulfite treatment, library preparation and sequencing (illumina Hiseq 4000, PE100) by the Beijing Genomics Institute (Shenzhen, China). Data analysis was performed according to Wang *et al*., 2016 [22].

### Locus-specific bisulfite sequencing

About 100 ng of genomic DNA was modified using the BisulFlash DNA Modification Kit (Epigentek, P-1026-050) according to the manufacturer’s instructions. An aliquot (1 μL) of bisulfite-treated DNA was used for PCR using the TaKaRa EpiTaq HS (for bisulfite-treated DNA) (Takara, R110A) with gene-specific primers (S1 Table) in a reaction volume of 20 μL. The PCR products were cloned into the pClone007 Blunt vector kit vector (TSINGKE, TSV-007B) and at least 20 independent clones of each sample were sequenced.

### Yeast two-hybrid assay

The CDSs of *TAH18*, *NBP35* and *GRXS17* were amplified by PCR and then subcloned into pGBK-T7 (Clontech, 630443). The CDSs of *DRE2*, *DRE2-3*, *DRE2*^*Δ6*^ and *DRE2*^*Δ75*^ were amplified by PCR and then subcloned into pGAD-T7 (Clontech, 630442). For protein interaction analysis, two combinatory constructs were transformed simultaneously into the yeast strain AH109 (Kept by our lab) and tested for Leu, Trp, Ade, and His auxotrophy according to the manufacturer’s protocols. The primers used for PCR are listed in S1 Table.

### Leptomycin B treatment

Leptomycin B treatment was performed as previously described [56]. Briefly, *pDRE2*::*DRE2-GFP* transgenic plants were grown vertically on 1/2 MS-medium plates for 5 days and then transferred to 3 mL of liquid 1/2 MS-medium (pH 5.7). After 48 h, Leptomycin B was added to a final concentration of 2.5 mM/L and incubated for 8–24 h.

### Fluorescence microscopy

FWA-GFP in the central cell and endosperm was detected using an Olympus BX53 fluorescence microscope equipped with an Olympus DP80 digital camera [57]. DRE2-GFP in the root was detected using the Nikon’s modular A1^+^/A1R^+^ confocal laser scanning microscope system (National Center for Protein Sciences at Peking University, Beijing).

### Subcellular fractionation and Western blot analysis

Tissues of 14-day-old *DRE2-HA* transgenic plants (about 1 g) were ground into fine powder and then suspended in 2 mL of Honda buffer (400 mM sucrose, 2.5% Ficoll, 5% Dextran T40, 25 mM Tris-HCl (pH 7.5), 10 mM MgCl_2_, 0.5% Triton X-100, 0.5 mM PMSF, 10 mM β-mercaptoethanol and protease inhibitor (1 tablet/100 mL)) The sample was centrifuged at 10,000 g for 20 min at 4°C. The homogenate was filtered through a double layer of Miracloth twice and then centrifuged at 1,500 g for 5 min at 4°C. The supernatant was further centrifuged at 10,000 g for 10 min at 4°C and the cytoplasmic fraction (supernatant) was collected. The pellet (nuclear fraction) was washed three times with Honda buffer and one time with PBS buffer (1 mM EDTA, 137 mM NaCl, 2.7 mM KCl, 10 mM Na_2_HPO_4_, 2 mM KH_2_PO_4_ (pH 7.4)) and then resuspended in 200 μL of glycerol buffer (20 mM Tris-HCl (pH 8.0), 75 mM NaCl, 0.5 mM EDTA, 0.85 mM DTT, 50% glycerol, 0.125 mM PMSF, 10 mM β-mercaptoethanol and protease inhibitor (1 tablet/100 mL)). Another 200 μL of nuclei lysis buffer (10 mM HEPES (pH 7.6), 1 mM DTT, 7.5 mM MgCl_2_, 0.2 mM EDTA, 300 mM NaCl, 1 M urea, 1% NP-40, and 0.5 mM PMSF, 10 mM β-mercaptoethanol and protease inhibitor (1 tablet/100 mL)) was added and the sample was vortexed gently. The mixture was incubated on ice for 5 min and then centrifuged at 13,200 g for 2 min at 4°C. The supernatant was collected as the nucleoplasmic fraction. The pellet was washed one time with PBS buffer and collected as the chromatin-associated fraction. The cytoplasmic marker HSP90 was detected by a rabbit anti-HSP90 polyclonal antibody (Santa Cruz Biotechnology, at-115). The nuclear marker Histone H3 was detected by a mouse anti-H3 monoclonal antibody (EASYBIO, BE7004-100). The cytoplasmic, nucleoplasmic and chromatin-associated protein markers AGO1 was detected by a rabbit anti-AGO1 polyclonal antibody (provided by Dr. Xiaoming Zhang, Institute of Zoology, CAS) [58]. DRE2-HA was detected by a mouse anti-HA monoclonal antibody (Sigma, H3663).

### Immunolocalization

Immunofluorescence staining was performed using leaves from 21-day-old *DRE2-HA* transgenic plant as previously described [59]. Nuclei preparations were incubated with a mouse anti-HA monoclonal antibody (Sigma, H3663) overnight at room temperature and then incubated with goat anti-mouse Alexa Fluor 488 secondary antibody (Invitrogen, A-11001) for 2 h at 37°C. DNA was counterstained using DAPI in Prolong Gold (Invitrogen, P36931). Pictures were taken using the Nikon’s modular A1^+^/A1R^+^ confocal laser scanning microscope system (National Center for Protein Sciences at Peking University, Beijing).

### RNA sequencing and data analysis

Total RNA samples were used to generate RNA libraries for deep sequencing (HiSeq XTEN, Illumina). Quality control was performed with **FastQC** (v0.11.5). Adapters and low quality reads were removed by **cutadapt** (v1.11). The trimmed and quality filtered clean reads were mapped to the *Arabidopsis* reference genome (TAIR10, https://www.arabidopsis.org) under the guide of annotation from Araport11 (https://www.araport.org) using **Tophat2** (v2.1.1). Reads were then sorted, indexed and compressed by **samtools** (v1.5) and bigwig files were generated by bam2wig.py with option–u (skip non-unique hit reads) from **RseQC** (v2.6.4). **Cuffdiff** (v2.2.1) was used to quantify the gene expression level and identify differentially expressed genes (DEGs) with option–u (use 'rescue method' for multi-reads). DEGs were filtered in **CummeRbund** (v2.16.0) under the criteria of FDR<0.05 and log2 fold change > 1. Gene ontology (GO) analysis was performed by **AgriGO** (http://bioinfo.cau.edu.cn/agriGO/analysis.php) with the default parameters. Heatmaps were generated by R package **ComplexHeatmap** (1.12.0). All statistical analysis analyses were performed in **R** (3.3.1).

### ChIP assay

Chromatin immunoprecipitation (ChIP) assays were performed according to published protocols [60,61] with minor modifications. Briefly, seedling tissues (about 1.5 g) were ground in liquid N_2_ and suspended in 20 mL of ChIP extraction buffer I (10 mM Tris-HCl (pH 8.0), 10 mM MgCl_2_, 400 mM sucrose, 0.1 mM PMSF, 1 mM DTT, protease inhibitor (1 tablet/100 mL)). The nuclei were fixed with 1% formaldehyde for 10 min at 4°C and then neutralized with 0.125 M glycine for 5 min. The nuclei were washed 4 times with ChIP extraction buffer II (10 mM Tris-HCl (pH 8.0), 10 mM MgCl_2_, 250 mM sucrose, 1% Triton X-100, 0.1 mM PMSF, 1 mM DTT, protease inhibitor (1 tablet/100 mL)) until they turned white, then layered on top of ChIP extraction buffer III (10 mM Tris-HCl (pH 8.0), 2 mM MgCl2, 1.7 M sucrose, 0.15% Triton-X100, 0.1 mM PMSF, 1 mM DTT, protease inhibitor (1 tablet/100 mL)) and centrifuged at 12000 rpm for 1 h. The purified nuclei were then suspended in nuclear lysis buffer (50 mM Tris-HCl (pH 8.0), 10 mM EDTA, 1% SDS), and sonicated for 28 cycles (30s on, 30s off). After centrifugation, the chromatin supernatant was diluted 10 times with dilution buffer (16.7 mM Tris-HCl (pH 8.0), 167 mM NaCl, 1.2 mM EDTA, 1.1% Triton X-100). An anti-GFP antibody (Abcam, ab290) was used for ChIP assays. After washing, the reversal of crosslinking and phenol chloroform extraction, the purified DNA was suspended in 50 μL of ddH_2_O and diluted 5 times. A 1 μL aliquot was used for quantitative PCR.

### Accession numbers

The RNA-seq data for Col-0 and *dre2-4* and the whole-genome bisulfite sequencing data for *dre2-4* was deposited at NCBI (SRP153123). The Col-0 and *ros1-4* whole-genome bisulfite sequencing data used were from NCBI (SRP119887).

## Supporting information

S1 Fig Generation of *dre2* hypomorphic mutants using the CRISPR/Cas9 system. (A-B) Positions of sgRNAs targeting *DRE2*. Gene (A) and protein (B) structure of *DRE2*. Positions of the three sgRNAs are marked by colored arrows. Sequencing results of T3 generation of *dre2* mutants carrying sgRNA-1 and sgRNA-2 are shown. (C) Phenotype of 28-day-old Col-0 and *dre2* mutants carrying sgRNA-1 and sgRNA-2. (D) Second biological replicate of Fig 1E. (E) The developmental phenotype of *dre2-4*. (F) Splicing variants of *DRE2* mRNA in *dre2-4*. Upper: sequence alignment of *DRE2* genomic DNA (DRE2g), wild type *DRE2* CDS, and mutant forms of *DRE2* CDS. Lower: a closer view of the wrong splicing site in *dre2-4*. The red box shows the original ‘AG’ splicing site, while the green box shows the alternatively selected ‘AG’ splicing site in *dre2-4*. Blue arrows indicate the positions of primers used for amplification of splicing variants of DRE2 mRNA in Fig 1C. (G) Amino acid sequence alignment of wild type and mutated forms of DRE2.
(PDF)

S2 Fig The *dre2-4* mutation does not affect *pFWA::ΔFWA-GFP* expression. ΔFWA-GFP expression in the central cell and in seeds 5 days after pollination in Col-0 and *dre2-4*.
(PDF)

S3 Fig The *dre2-4* mutant is sensitive to MMS treatment. (A) Phenotype of 14-day-old Col-0 and *dre2-4* seedlings with 0 and 60 ppm of MMS. (B) Relative expression levels of the indicated genes in the indicated genotypes as determined by RT-qPCR. Data are presented as mean ± SD of four technical replicates. Asterisks indicate two-tailed Student’s *t*-test, *P < 0.05, **P < 0.01. Results from the second biological replicate are shown in S4C Fig.
(PDF)

S4 Fig Overexpression of *DRE2* rescued the upregulation of DNA Damage Response genes and cyclin genes in *dre2-4*. (A-C) Relative expression levels of the indicated genes in the indicated genotypes as determined by RT-qPCR. Data are presented as mean ± SD of four technical replicates. Asterisks indicate two-tailed Student’s *t*-test, *P < 0.05, **P < 0.01.
(PDF)

S5 Fig DNA methylome analysis by whole-genome bisulfite sequencing. (A) The number of differentially methylated regions (DMRs) identified in *dre2-4* and the ratio of hyper-DMRs overlapping with *ros1-4*. (B) Composition of the hypermethylated loci in *dre2-4* and *ros1-4*. (C) Snapshot in the Integrated Genome Browser showing the DNA methylation levels of the *ROS1* promoter in different genotypes. The specific region important for the regulation of *ROS1* expression is highlighted with red box. (D) Relative expression levels of *ROS1* in the indicated genotypes as determined by RT-qPCR. Data are presented as mean ± SD of four technical replicates. Asterisks indicate two-tailed Student’s *t*-test, *P < 0.05, **P < 0.01.
(PDF)

S6 Fig Effects of *dre2-4* on RNA transcript levels as determined by RNA-seq. (A-B) Gene Ontology analysis of significantly upregulated (A) and downregulated (B) genes in *dre2-4*. The lengths of bars represent statistical values of gene enrichment in the indicated biological processes. The biological processes listed are significantly (FDR<0.05) enriched. (C) Heatmap showing log_2_ (FPKM+1) values of genes involved in DDR, cell cycle and DNA replication in the six samples (three replicates of Col-0 and three replicates of *dre2-4*). Color keys are on the right. (D) Heatmap showing log_2_ (FPKM+1) values of genes involved in auxin response in the six samples (three replicates of Col-0 and three replicates of *dre2-4*). Color keys are on the right.
(PDF)

S7 Fig Primary root length inhibition is alleviated in *dre2* mutants under IAA treatment. (A) Primary root lengths of the indicated genotypes with or without 10 μM IAA treatment. (B) Relative primary root lengths of the indicated genotypes showing the inhibition of primary root growth by IAA. The ratios of primary root length after IAA treatment versus that without IAA treatment were calculated.
(PDF)

S8 Fig Localization of DRE2-GFP. (A) DRE2-GFP expression in the differentiation zone of root without or with Leptomycin B treatment. (B) DRE2-GFP expression in the meristematic zone of root.
(PDF)

S9 Fig Original Western blot data. (PDF)

S1 Table Primers used in this study. (PDF)

S1 Dataset List of differentially methylated regions in *dre2-4* and *ros1-4*. (XLSX)

S2 Dataset List of differentially expressed genes in *dre2-4* detected by RNA-seq. (XLSX)

## References

[1] Lill R. Function and biogenesis of iron-sulphur proteins. Nature. 2009;460(7257):831–8. 10.1038/nature08301 19675643

[2] Beinert H, Holm RH, Munck E. Iron-sulfur clusters: nature's modular, multipurpose structures. Science. 1997;277(5326):653–9. 9235882 10.1126/science.277.5326.653

[3] Lill R, Muhlenhoff U. Iron-sulfur protein biogenesis in eukaryotes: components and mechanisms. Annu Rev Cell Dev Biol. 2006;22:457–86. 10.1146/annurev.cellbio.22.010305.104538 16824008

[4] Balk J, Pilon M. Ancient and essential: the assembly of iron-sulfur clusters in plants. Trends Plant Sci. 2011;16(4):218–26. 10.1016/j.tplants.2010.12.006 21257336

[5] Netz DJ, Mascarenhas J, Stehling O, Pierik AJ, Lill R. Maturation of cytosolic and nuclear iron-sulfur proteins. Trends Cell Biol. 2014;24(5):303–12. 10.1016/j.tcb.2013.11.005 24314740

[6] Couturier J, Touraine B, Briat JF, Gaymard F, Rouhier N. The iron-sulfur cluster assembly machineries in plants: current knowledge and open questions. Front Plant Sci. 2013;4:259 10.3389/fpls.2013.00259 23898337 PMC3721309

[7] Lill R, Hoffmann B, Molik S, Pierik AJ, Rietzschel N, Stehling O, et al The role of mitochondria in cellular iron-sulfur protein biogenesis and iron metabolism. Biochim Biophys Acta. 2012;1823(9):1491–508. 10.1016/j.bbamcr.2012.05.009 22609301

[8] Schaedler TA, Thornton JD, Kruse I, Schwarzlander M, Meyer AJ, van Veen HW, et al A conserved mitochondrial ATP-binding cassette transporter exports glutathione polysulfide for cytosolic metal cofactor assembly. J Biol Chem. 2014;289(34):23264–74. 10.1074/jbc.M114.553438 25006243 PMC4156053

[9] Roy A, Solodovnikova N, Nicholson T, Antholine W, Walden WE. A novel eukaryotic factor for cytosolic Fe-S cluster assembly. EMBO J. 2003;22(18):4826–35. 10.1093/emboj/cdg455 12970194 PMC212722

[10] Hausmann A, Aguilar Netz DJ, Balk J, Pierik AJ, Muhlenhoff U, Lill R. The eukaryotic P loop NTPase Nbp35: an essential component of the cytosolic and nuclear iron-sulfur protein assembly machinery. Proc Natl Acad Sci U S A. 2005;102(9):3266–71. 10.1073/pnas.0406447102 15728363 PMC552912

[11] Netz DJ, Pierik AJ, Stumpfig M, Muhlenhoff U, Lill R. The Cfd1-Nbp35 complex acts as a scaffold for iron-sulfur protein assembly in the yeast cytosol. Nat Chem Biol. 2007;3(5):278–86. 10.1038/nchembio872 17401378

[12] Balk J, Aguilar Netz DJ, Tepper K, Pierik AJ, Lill R. The essential WD40 protein Cia1 is involved in a late step of cytosolic and nuclear iron-sulfur protein assembly. Mol Cell Biol. 2005;25(24):10833–41. 10.1128/MCB.25.24.10833-10841.2005 16314508 PMC1316972

[13] Vo AT, Fleischman NM, Marquez MD, Camire EJ, Esonwune SU, Grossman JD, et al Defining the domains of Cia2 required for its essential function *in vivo* and *in vitro*. Metallomics. 2017;9(11):1645–54. 10.1039/c7mt00181a 29057997

[14] Stehling O, Vashisht AA, Mascarenhas J, Jonsson ZO, Sharma T, Netz DJ, et al MMS19 assembles iron-sulfur proteins required for DNA metabolism and genomic integrity. Science. 2012;337(6091):195–9. 10.1126/science.1219723 22678362 PMC3420340

[15] Gari K, Leon Ortiz AM, Borel V, Flynn H, Skehel JM, Boulton SJ. MMS19 links cytoplasmic iron-sulfur cluster assembly to DNA metabolism. Science. 2012;337(6091):243–5. 10.1126/science.1219664 22678361

[16] Balk J, Pierik AJ, Netz DJ, Muhlenhoff U, Lill R. The hydrogenase-like Nar1p is essential for maturation of cytosolic and nuclear iron-sulphur proteins. EMBO J. 2004;23(10):2105–15. 10.1038/sj.emboj.7600216 15103330 PMC424389

[17] Bernard DG, Cheng Y, Zhao Y, Balk J. An allelic mutant series of *ATM3* reveals its key role in the biogenesis of cytosolic iron-sulfur proteins in *Arabidopsis*. Plant Physiol. 2009;151(2):590–602. 10.1104/pp.109.143651 19710232 PMC2754654

[18] Zuo J, Wu Z, Li Y, Shen Z, Feng X, Zhang M, et al Mitochondrial ABC Transporter ATM3 Is Essential for Cytosolic Iron-Sulfur Cluster Assembly. Plant Physiol. 2017;173(4):2096–109. 10.1104/pp.16.01760 28250070 PMC5373059

[19] Luo D, Bernard DG, Balk J, Hai H, Cui X. The DUF59 family gene *AE7* acts in the cytosolic iron-sulfur cluster assembly pathway to maintain nuclear genome integrity in *Arabidopsis*. Plant Cell. 2012;24(10):4135–48. 10.1105/tpc.112.102608 23104832 PMC3517241

[20] Duan CG, Wang X, Tang K, Zhang H, Mangrauthia SK, Lei M, et al MET18 Connects the Cytosolic Iron-Sulfur Cluster Assembly Pathway to Active DNA Demethylation in Arabidopsis. PLoS Genet. 2015;11(10):e1005559 10.1371/journal.pgen.1005559 26492035 PMC4619598

[21] Han YF, Huang HW, Li L, Cai T, Chen S, He XJ. The Cytosolic Iron-Sulfur Cluster Assembly Protein MMS19 Regulates Transcriptional Gene Silencing, DNA Repair, and Flowering Time in Arabidopsis. PLoS One. 2015;10(6):e0129137 10.1371/journal.pone.0129137 26053632 PMC4459967

[22] Wang X, Li Q, Yuan W, Cao Z, Qi B, Kumar S, et al The cytosolic Fe-S cluster assembly component MET18 is required for the full enzymatic activity of ROS1 in active DNA demethylation. Sci Rep. 2016;6:26443 10.1038/srep26443 27193999 PMC4872223

[23] Nakamura M, Buzas DM, Kato A, Fujita M, Kurata N, Kinoshita T. The role of *Arabidopsis thaliana* NAR1, a cytosolic iron-sulfur cluster assembly component, in gametophytic gene expression and oxidative stress responses in vegetative tissue. New Phytol. 2013;199(4):925–35. 10.1111/nph.12350 23734982

[24] Zhang Y, Lyver ER, Nakamaru-Ogiso E, Yoon H, Amutha B, Lee DW, et al Dre2, a conserved eukaryotic Fe/S cluster protein, functions in cytosolic Fe/S protein biogenesis. Mol Cell Biol. 2008;28(18):5569–82. 10.1128/MCB.00642-08 18625724 PMC2546940

[25] Netz DJ, Stumpfig M, Dore C, Muhlenhoff U, Pierik AJ, Lill R. Tah18 transfers electrons to Dre2 in cytosolic iron-sulfur protein biogenesis. Nat Chem Biol. 2010;6(10):758–65. 10.1038/nchembio.432 20802492

[26] Netz DJ, Genau HM, Weiler BD, Bill E, Pierik AJ, Lill R. The conserved protein Dre2 uses essential [2Fe-2S] and [4Fe-4S] clusters for its function in cytosolic iron-sulfur protein assembly. Biochem J. 2016;473(14):2073–85. 10.1042/BCJ20160416 27166425

[27] Banci L, Ciofi-Baffoni S, Gajda K, Muzzioli R, Peruzzini R, Winkelmann J. N-terminal domains mediate [2Fe-2S] cluster transfer from glutaredoxin-3 to anamorsin. Nat Chem Biol. 2015;11(10):772–8. 10.1038/nchembio.1892 26302480

[28] Banci L, Camponeschi F, Ciofi-Baffoni S, Muzzioli R. Elucidating the Molecular Function of Human BOLA2 in GRX3-Dependent Anamorsin Maturation Pathway. J Am Chem Soc. 2015;137(51):16133–43. 10.1021/jacs.5b10592 26613676

[29] Varadarajan J, Guilleminot J, Saint-Jore-Dupas C, Piegu B, Chaboute ME, Gomord V, et al *ATR3* encodes a diflavin reductase essential for *Arabidopsis* embryo development. New Phytol. 2010;187(1):67–82. 10.1111/j.1469-8137.2010.03254.x 20406405

[30] Inigo S, Durand AN, Ritter A, Le Gall S, Termathe M, Klassen R, et al Glutaredoxin GRXS17 Associates with the Cytosolic Iron-Sulfur Cluster Assembly Pathway. Plant Physiol. 2016;172(2):858–73. 10.1104/pp.16.00261 27503603 PMC5047072

[31] Bastow EL, Bych K, Crack JC, Le Brun NE, Balk J. NBP35 interacts with DRE2 in the maturation of cytosolic iron-sulphur proteins in *Arabidopsis thaliana*. Plant J. 2017;89(3):590–600. 10.1111/tpj.13409 27801963 PMC5324674

[32] Buzas DM, Nakamura M, Kinoshita T. Epigenetic role for the conserved Fe-S cluster biogenesis protein AtDRE2 in *Arabidopsis thaliana*. Proc Natl Acad Sci U S A. 2014;111(37):13565–70. 10.1073/pnas.1404058111 25197096 PMC4169955

[33] Song G, Cheng C, Li Y, Shaw N, Xiao ZC, Liu ZJ. Crystal structure of the N-terminal methyltransferase-like domain of anamorsin. Proteins. 2014;82(6):1066–71. 10.1002/prot.24443 24123282

[34] Doucet-Chabeaud G GC, Brutesco C, de Murcia G, Kazmaier M. Ionising radiation induces the expression of *PARP-1* and *PARP-2* genes in *Arabidopsis*. Mol Genet Genomics. 2001;265(6):954–63. 11523787 10.1007/s004380100506

[35] Strzalka W, Ziemienowicz A. Proliferating cell nuclear antigen (PCNA): a key factor in DNA replication and cell cycle regulation. Ann Bot. 2011;107(7):1127–40. 10.1093/aob/mcq243 21169293 PMC3091797

[36] Reidt W, Wurz R, Wanieck K, Chu HH, Puchta H. A homologue of the breast cancer-associated gene *BARD1* is involved in DNA repair in plants. EMBO J. 2006;25(18):4326–37. 10.1038/sj.emboj.7601313 16957774 PMC1570427

[37] Gong Z, Morales-Ruiz T, Ariza RR, Roldan-Arjona T, David L, Zhu JK. *ROS1*, a repressor of transcriptional gene silencing in *Arabidopsis*, encodes a DNA glycosylase/lyase. Cell. 2002;111(6):803–14. 12526807 10.1016/s0092-8674(02)01133-9

[38] Lei M, Zhang H, Julian R, Tang K, Xie S, Zhu JK. Regulatory link between DNA methylation and active demethylation in *Arabidopsis*. Proc Natl Acad Sci U S A. 2015;112(11):3553–7. 10.1073/pnas.1502279112 25733903 PMC4371987

[39] Williams BP, Pignatta D, Henikoff S, Gehring M. Methylation-sensitive expression of a DNA demethylase gene serves as an epigenetic rheostat. PLoS Genet. 2015;11(3):e1005142 10.1371/journal.pgen.1005142 25826366 PMC4380477

[40] Banci L, Bertini I, Ciofi-Baffoni S, Boscaro F, Chatzi A, Mikolajczyk M, et al Anamorsin is a [2Fe-2S] cluster-containing substrate of the Mia40-dependent mitochondrial protein trapping machinery. Chem Biol. 2011;18(6):794–804. 10.1016/j.chembiol.2011.03.015 21700214

[41] Lavenus J, Goh T, Roberts I, Guyomarc'h S, Lucas M, De Smet I, et al Lateral root development in *Arabidopsis*: fifty shades of auxin. Trends Plant Sci. 2013;18(8):450–8. 10.1016/j.tplants.2013.04.006 23701908

[42] Teale WD, Paponov IA, Palme K. Auxin in action: signalling, transport and the control of plant growth and development. Nat Rev Mol Cell Biol. 2006;7(11):847–59. 10.1038/nrm2020 16990790

[43] Smith I, Greenside PG, Natoli T, Lahr DL, Wadden D, Tirosh I, et al Evaluation of RNAi and CRISPR technologies by large-scale gene expression profiling in the Connectivity Map. PLoS Biol. 2017;15(11):e2003213 10.1371/journal.pbio.2003213 29190685 PMC5726721

[44] Netz DJ, Stith CM, Stumpfig M, Kopf G, Vogel D, Genau HM, et al Eukaryotic DNA polymerases require an iron-sulfur cluster for the formation of active complexes. Nat Chem Biol. 2011;8(1):125–32. 10.1038/nchembio.721 22119860 PMC3241888

[45] Klinge S, Hirst J, Maman JD, Krude T, Pellegrini L. An iron-sulfur domain of the eukaryotic primase is essential for RNA primer synthesis. Nat Struct Mol Biol. 2007;14(9):875–7. 10.1038/nsmb1288 17704817 PMC2268749

[46] Vaithiyalingam S, Warren EM, Eichman BF, Chazin WJ. Insights into eukaryotic DNA priming from the structure and functional interactions of the 4Fe-4S cluster domain of human DNA primase. Proc Natl Acad Sci U S A. 2010;107(31):13684–9. 10.1073/pnas.1002009107 20643958 PMC2922289

[47] Rudolf J, Makrantoni V, Ingledew WJ, Stark MJ, White MF. The DNA repair helicases XPD and FancJ have essential iron-sulfur domains. Mol Cell. 2006;23(6):801–8. 10.1016/j.molcel.2006.07.019 16973432

[48] Barber LJ, Youds JL, Ward JD, McIlwraith MJ, O'Neil NJ, Petalcorin MI, et al RTEL1 maintains genomic stability by suppressing homologous recombination. Cell. 2008;135(2):261–71. 10.1016/j.cell.2008.08.016 18957201 PMC3726190

[49] Banci L, Bertini I, Calderone V, Ciofi-Baffoni S, Giachetti A, Jaiswal D, et al Molecular view of an electron transfer process essential for iron-sulfur protein biogenesis. Proc Natl Acad Sci U S A. 2013;110(18):7136–41. 10.1073/pnas.1302378110 23596212 PMC3645582

[50] Cheng NH, Liu JZ, Liu X, Wu Q, Thompson SM, Lin J, et al *Arabidopsis* monothiol glutaredoxin, AtGRXS17, is critical for temperature-dependent postembryonic growth and development via modulating auxin response. J Biol Chem. 2011;286(23):20398–406. 10.1074/jbc.M110.201707 21515673 PMC3121514

[51] Wang ZP, Xing HL, Dong L, Zhang HY, Han CY, Wang XC, et al Egg cell-specific promoter-controlled CRISPR/Cas9 efficiently generates homozygous mutants for multiple target genes in *Arabidopsis* in a single generation. Genome Biol. 2015;16:144 10.1186/s13059-015-0715-0 26193878 PMC4507317

[52] Clough SJ, Bent AF. Floral dip: a simplified method for *Agrobacterium*‐mediated transformation of *Arabidopsis thaliana*. Plant J.1998;16(6):735–43. 10069079 10.1046/j.1365-313x.1998.00343.x

[53] Seo M, Akaba S, Oritani T, Delarue M, Bellini C, Caboche M, et al Higher activity of an aldehyde oxidase in the auxin-overproducing *superroot1* mutant of Arabidopsis thaliana. Plant Physiol. 1998;116(2):687–93. 9489015 10.1104/pp.116.2.687 PMC35127

[54] Koshiba T, Saito E, Ono N, Yamamoto N, Sato M. Purification and Properties of Flavin- and Molybdenum-Containing Aldehyde Oxidase from Coleoptiles of Maize. Plant Physiol. 1996;110(3):781–9. 12226218 10.1104/pp.110.3.781 PMC157777

[55] Seo M, Peeters AJ, Koiwai H, Oritani T, Marion-Poll A, Zeevaart JA, et al The Arabidopsis aldehyde oxidase 3 (AAO3) gene product catalyzes the final step in abscisic acid biosynthesis in leaves. Proc Natl Acad Sci U S A. 2000;97(23):12908–13. 10.1073/pnas.220426197 11050171 PMC18863

[56] Bologna NG, Iselin R, Abriata LA, Sarazin A, Pumplin N, Jay F, et al Nucleo-cytosolic Shuttling of ARGONAUTE1 Prompts a Revised Model of the Plant MicroRNA Pathway. Mol Cell. 2018;69(4):709–19 e5. 10.1016/j.molcel.2018.01.007 29398448

[57] Li Y, Cordoba-Canero D, Qian W, Zhu X, Tang K, Zhang H, et al An AP endonuclease functions in active DNA dimethylation and gene imprinting in *Arabidopsis*. PLoS Genet. 2015;11(1):e1004905 10.1371/journal.pgen.1004905 25569774 PMC4287435

[58] Liu C, Xin Y, Xu L, Cai Z, Xue Y, Liu Y, et al *Arabidopsis* ARGONAUTE 1 Binds Chromatin to Promote Gene Transcription in Response to Hormones and Stresses. Dev Cell. 2018;44(3):348–61 e7. 10.1016/j.devcel.2017.12.002 29290588

[59] Martinez-Macias MI, Qian W, Miki D, Pontes O, Liu Y, Tang K, et al A DNA 3' phosphatase functions in active DNA demethylation in *Arabidopsis*. Mol Cell. 2012;45(3):357–70. 10.1016/j.molcel.2011.11.034 22325353 PMC3278721

[60] Gendrel A-V, Lippman Z, Martienssen R, Colot V. Profiling histone modification patterns in plants using genomic tiling microarrays. Nat Methods. 2005;2:213 10.1038/nmeth0305-213 16163802

[61] Bowler C, Benvenuto G, Laflamme P, Molino D, Probst AV, Tariq M, et al Chromatin techniques for plant cells. Plant J. 2004;39(5):776–89. 10.1111/j.1365-313X.2004.02169.x 15315638

