# Supplementary material for: Canonical cytosolic iron-sulfur cluster assembly and non-canonical functions of DRE2 in Arabidopsis
Source: PLoS Genet. 2019 Apr 29;15(4):e1008094. doi: 10.1371/journal.pgen.1008094 (PMC6508740; doi:10.1371/journal.pgen.1008094)
Supplement: S1 Fig — (A-B) Positions of sgRNAs targeting DRE2. Gene (A) and protein (B) structure of DRE2. Positions of the three sgRNAs are marked by colored arrows. Sequencing results of T3 generation of dre2 mutants carrying sgRNA-1 and sgRNA-2 are shown. (C) Phenotype of 28-day-old Col-0 and dre2 mutants carrying sgRNA-1 and sgRNA-2. (D) Second biological replicate of Fig 1E. (E) The developmental phenotype of dre2-4. (F) Splicing variants of DRE2 mRNA in dre2-4. Upper: sequence alignment of DRE2 genomic DNA (DRE2g), wild type DRE2 CDS, and mutant forms of DRE2 CDS. Lower: a closer view of the wrong splicing site in dre2-4. The red box shows the original ‘AG’ splicing site, while the green box shows the alternatively selected ‘AG’ splicing site in dre2-4. Blue arrows indicate the positions of primers used for amplification of splicing variants of DRE2 mRNA in Fig 1C. (G) Amino acid sequence alignment of wild type and mutated forms of DRE2. (PDF) [file pgen.1008094.s001.pdf]

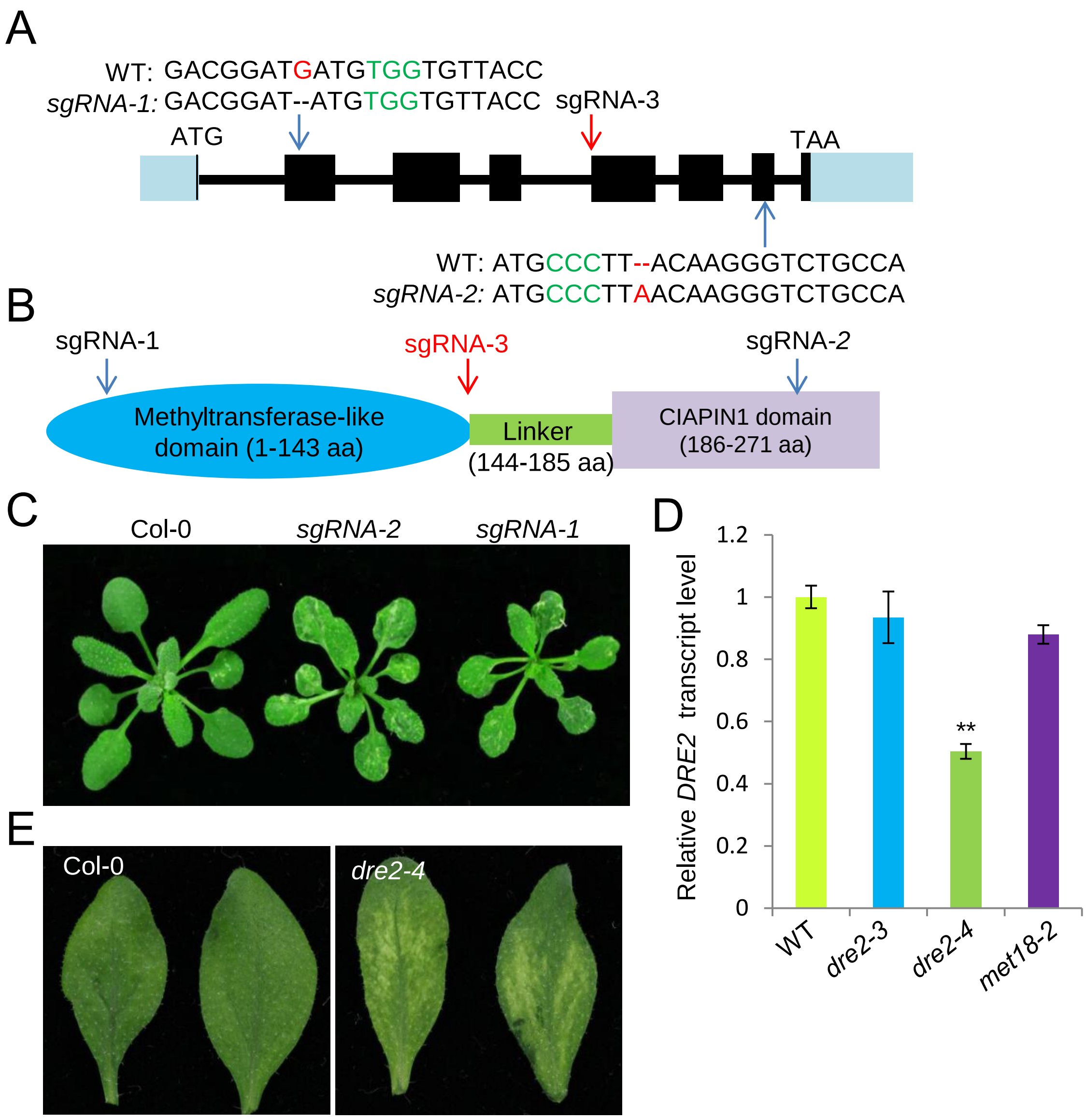

S1 Fig. Generation of *dre2* hypomorphic mutants using the CRISPR/Cas9 system.

(A-B) Positions of sgRNAs targeting *DRE2*. Gene (A) and protein (B) structure of *DRE2*. Positions of the three sgRNAs are marked by colored arrows. Sequencing results of T3 generation of *dre2* mutants carrying sgRNA-1 and sgRNA-2 are shown. (C) Phenotype of 28-day-old Col-0 and *dre2* mutants carrying sgRNA-1 and sgRNA-2. (D) Second biological replicate of Fig 1E. (E) The developmental phenotype of *dre2-4*. (F) Splicing variants of *DRE2* mRNA in *dre2-4*. Upper: sequence alignment of *DRE2* genomic DNA (DRE2g), wild type *DRE2* CDS, and mutant forms of *DRE2* CDS. Lower: a closer view of the wrong splicing site in *dre2-4*. The red box shows the original ‘AG’ splicing site, while the green box shows the alternatively selected ‘AG’ splicing site in *dre2-4*. Blue arrows indicate the positions of primers used for amplification of splicing variants of DRE2 mRNA in Fig 1C. (G) Amino acid sequence alignment of wild type and mutated forms of DRE2.

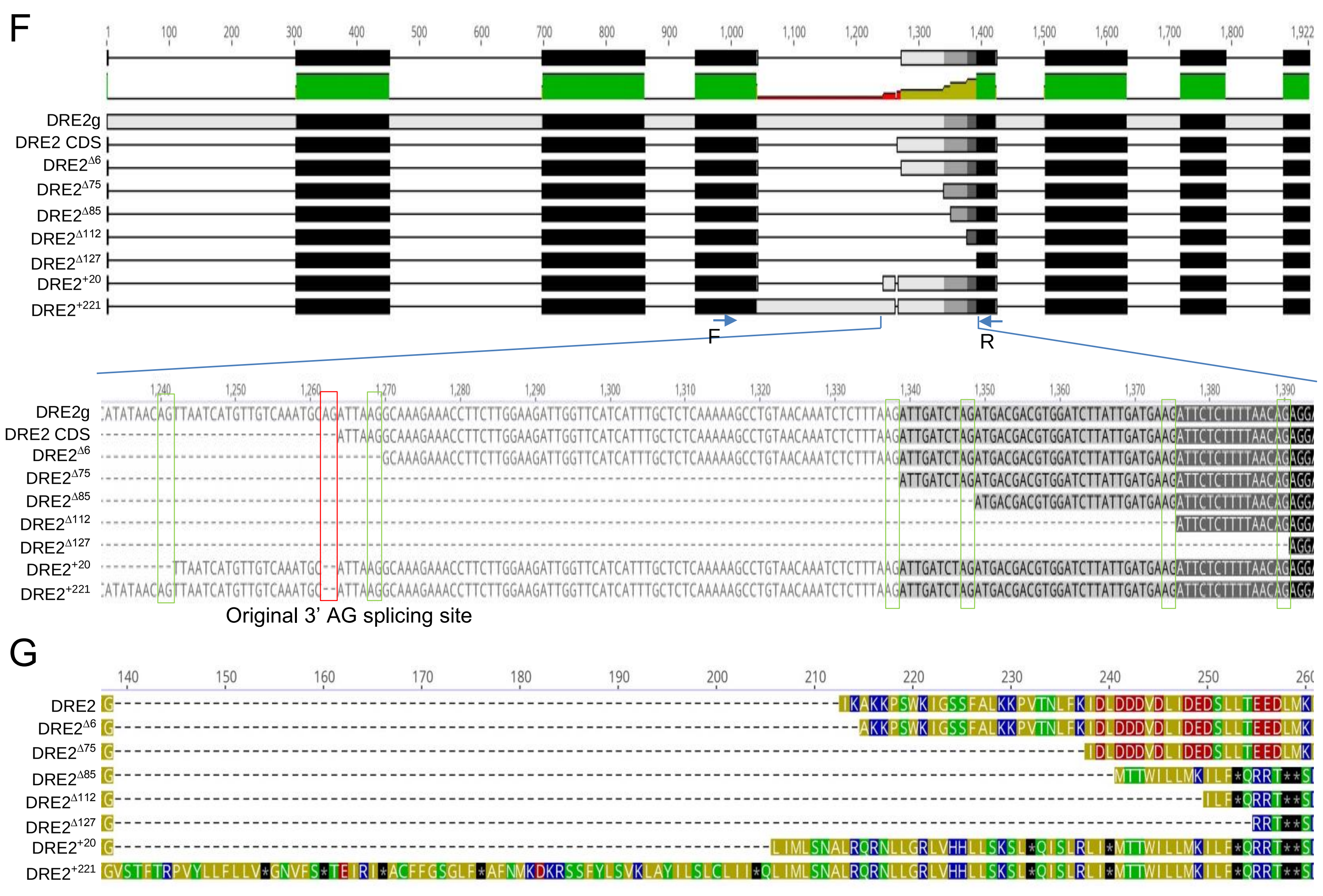
F
DRE2g
DRE2 CDS
DRE2Δ6
DRE2Δ75
DRE2Δ85
DRE2Δ112
DRE2Δ127
DRE2+20
DRE2+221
F
R
Original 3' AG splicing site
G
DRE2
DRE2Δ6
DRE2Δ75
DRE2Δ85
DRE2Δ112
DRE2Δ127
DRE2+20
DRE2+221

S1 Fig
