# Supplementary material for: Canonical cytosolic iron-sulfur cluster assembly and non-canonical functions of DRE2 in Arabidopsis
Source: PLoS Genet. 2019 Apr 29;15(4):e1008094. doi: 10.1371/journal.pgen.1008094 (PMC6508740; doi:10.1371/journal.pgen.1008094)
Supplement: S2 Fig — ΔFWA-GFP expression in the central cell and in seeds 5 days after pollination in Col-0 and dre2-4. (PDF) [file pgen.1008094.s002.pdf]

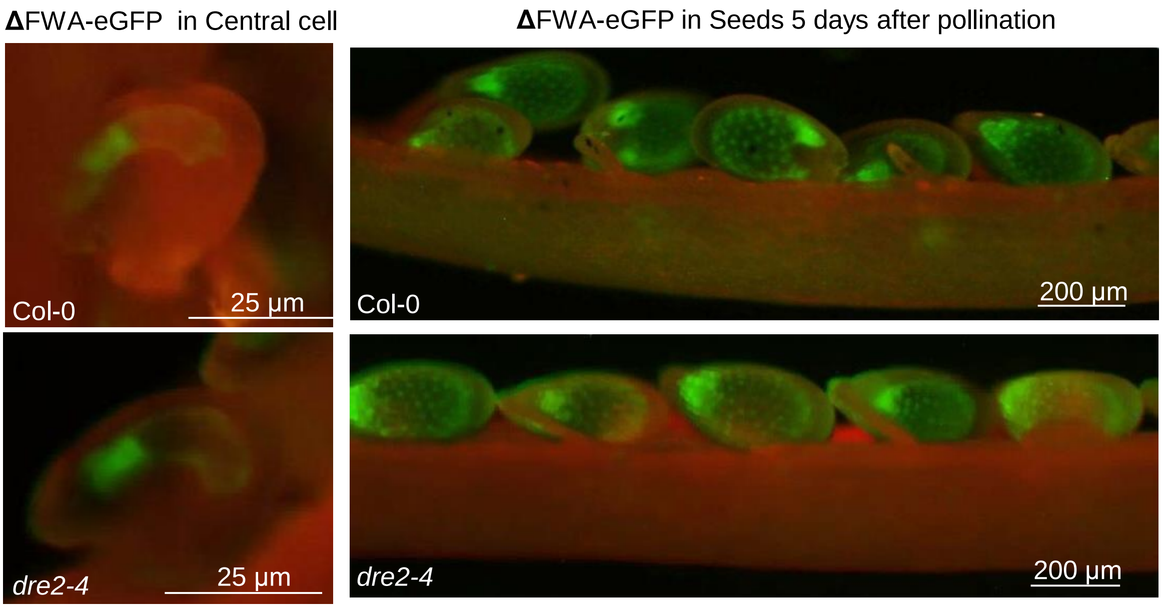

S2 Fig. The *dre2-4* mutation does not affect *pFWA::ΔFWA-GFP* expression.

ΔFWA-GFP expression in the central cell and in seeds 5 days after pollination in Col-0 and *dre2-4*.
