# Supplementary material for: Canonical cytosolic iron-sulfur cluster assembly and non-canonical functions of DRE2 in Arabidopsis
Source: PLoS Genet. 2019 Apr 29;15(4):e1008094. doi: 10.1371/journal.pgen.1008094 (PMC6508740; doi:10.1371/journal.pgen.1008094)
Supplement: S3 Fig — (A) Phenotype of 14-day-old Col-0 and dre2-4 seedlings with 0 and 60 ppm of MMS. (B) Relative expression levels of the indicated genes in the indicated genotypes as determined by RT-qPCR. Data are presented as mean ± SD of four technical replicates. Asterisks indicate two-tailed Student’s t-test, *P < 0.05, **P < 0.01. Results from the second biological replicate are shown in S4C Fig. (PDF) [file pgen.1008094.s003.pdf]

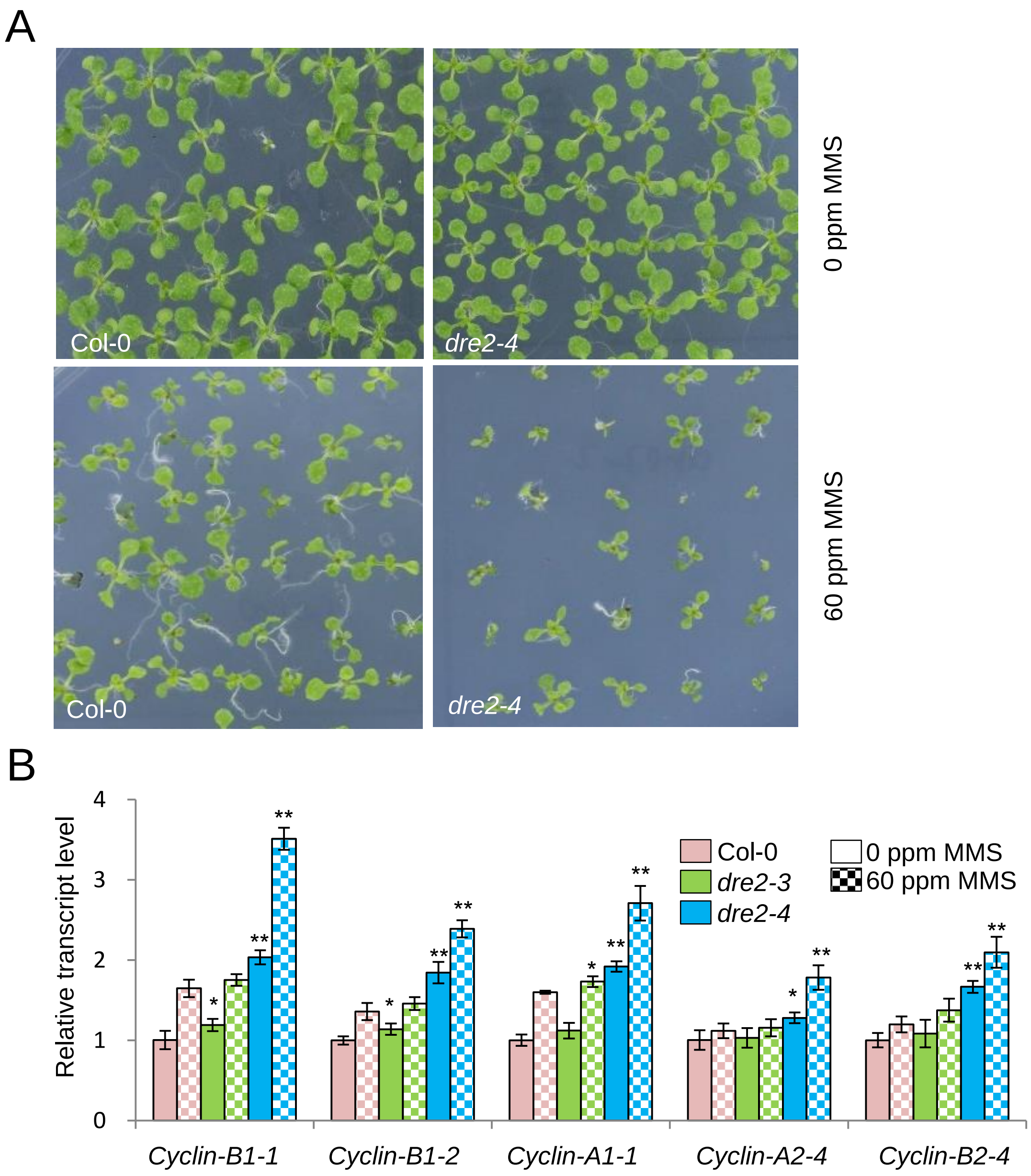

S3 Fig. The *dre2-4* mutant is sensitive to MMS treatment.

(A) Phenotype of 14-day-old Col-0 and *dre2-4* seedlings with 0 and 60 ppm of MMS. (B) Relative expression levels of the indicated genes in the indicated genotypes as determined by RT-qPCR. Data are presented as mean ± SD of four technical replicates. Asterisks indicate two-tailed Student's *t*-test, **P* < 0.05, ***P* < 0.01. Results from the second biological replicate are shown in S4C Fig.
