# Supplementary material for: Canonical cytosolic iron-sulfur cluster assembly and non-canonical functions of DRE2 in Arabidopsis
Source: PLoS Genet. 2019 Apr 29;15(4):e1008094. doi: 10.1371/journal.pgen.1008094 (PMC6508740; doi:10.1371/journal.pgen.1008094)
Supplement: S4 Fig — (A-C) Relative expression levels of the indicated genes in the indicated genotypes as determined by RT-qPCR. Data are presented as mean ± SD of four technical replicates. Asterisks indicate two-tailed Student’s t-test, *P < 0.05, **P < 0.01. (PDF) [file pgen.1008094.s004.pdf]

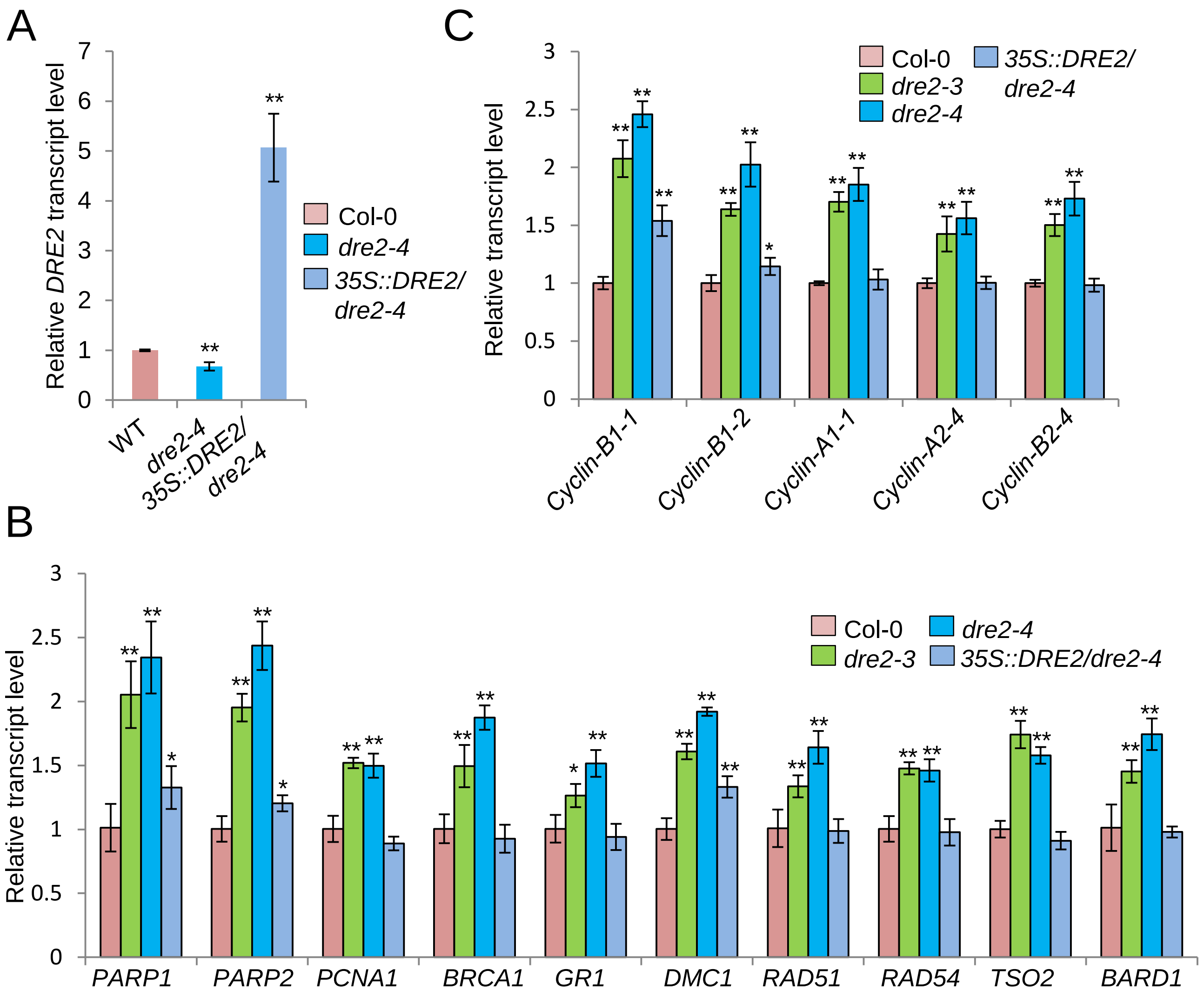

S4 Fig. Overexpression of *DRE2* rescued the upregulation of DNA Damage Response genes and cyclin genes in *dre2-4* .

(A-C) Relative expression levels of the indicated genes in the indicated genotypes as determined by RT-qPCR. Data are presented as mean ± SD of four technical replicates. Asterisks indicate two-tailed Student's *t*-test, **P* < 0.05, ***P* < 0.01.
