# Supplementary material for: Canonical cytosolic iron-sulfur cluster assembly and non-canonical functions of DRE2 in Arabidopsis
Source: PLoS Genet. 2019 Apr 29;15(4):e1008094. doi: 10.1371/journal.pgen.1008094 (PMC6508740; doi:10.1371/journal.pgen.1008094)
Supplement: S5 Fig — (A) The number of differentially methylated regions (DMRs) identified in dre2-4 and the ratio of hyper-DMRs overlapping with ros1-4. (B) Composition of the hypermethylated loci in dre2-4 and ros1-4. (C) Snapshot in the Integrated Genome Browser showing the DNA methylation levels of the ROS1 promoter in different genotypes. The specific region important for the regulation of ROS1 expression is highlighted with red box. (D) Relative expression levels of ROS1 in the indicated genotypes as determined by RT-qPCR. Data are presented as mean ± SD of four technical replicates. Asterisks indicate two-tailed Student’s t-test, *P < 0.05, **P < 0.01. (PDF) [file pgen.1008094.s005.pdf]

A

Genotype	DMRs	Hyper-DMRs	Hypo-DMRs	Hyper-DMRs overlapped with ros1-4
ros1-4	4,293	4,164	129	-
dre2-4	1,459	951	508	66.6%

B

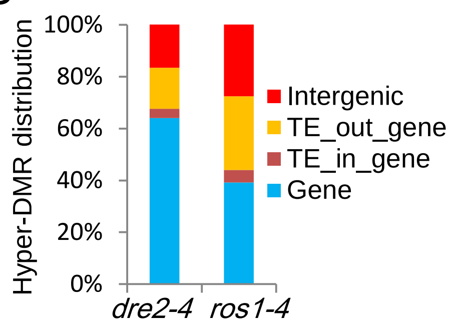

C

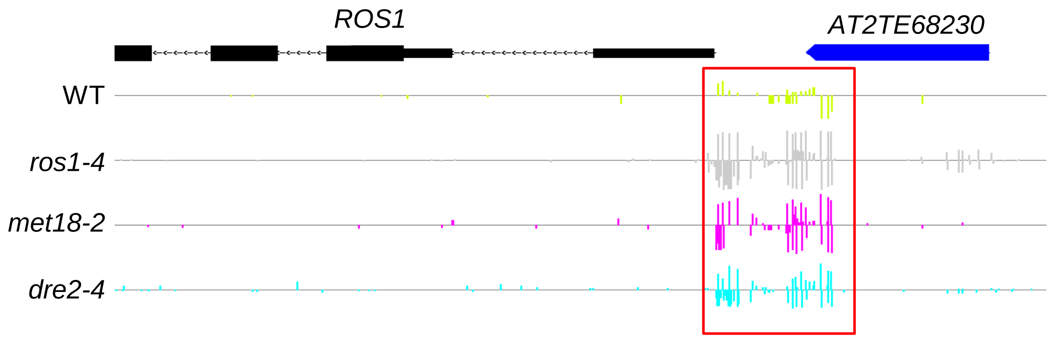

D

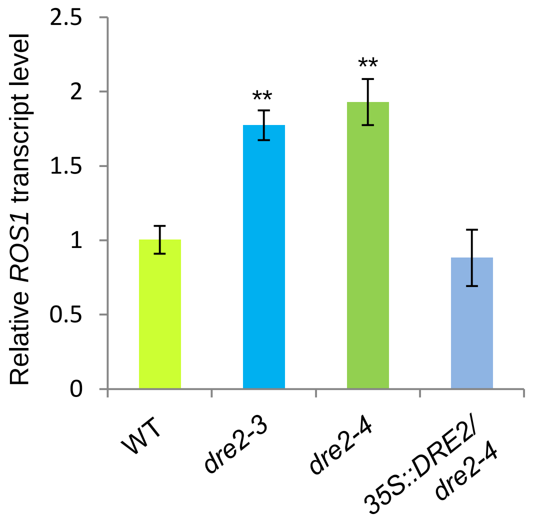

S5 Fig. DNA methylome analysis by whole-genome bisulfite sequencing.
(A) The number of differentially methylated regions (DMRs) identified in *dre2-4* and the ratio of hyper-DMRs overlapping with *ros1-4*. (B) Composition of the hypermethylated loci in *dre2-4* and *ros1-4*. (C) Snapshot in the Integrated Genome Browser showing the DNA methylation levels of the *ROS1* promoter in different genotypes. The specific region important for the regulation of *ROS1* expression is highlighted with red box. (D) Relative expression levels of *ROS1* in the indicated genotypes as determined by RT-qPCR. Data are presented as mean ± SD of four technical replicates. Asterisks indicate two-tailed Student's *t*-test, *$P < 0.05$, **$P < 0.01$.
