# Supplementary material for: Canonical cytosolic iron-sulfur cluster assembly and non-canonical functions of DRE2 in Arabidopsis
Source: PLoS Genet. 2019 Apr 29;15(4):e1008094. doi: 10.1371/journal.pgen.1008094 (PMC6508740; doi:10.1371/journal.pgen.1008094)
Supplement: S6 Fig — (A-B) Gene Ontology analysis of significantly upregulated (A) and downregulated (B) genes in dre2-4. The lengths of bars represent statistical values of gene enrichment in the indicated biological processes. The biological processes listed are significantly (FDR<0.05) enriched. (C) Heatmap showing log2 (FPKM+1) values of genes involved in DDR, cell cycle and DNA replication in the six samples (three replicates of Col-0 and three replicates of dre2-4). Color keys are on the right. (D) Heatmap showing log2 (FPKM+1) values of genes involved in auxin response in the six samples (three replicates of Col-0 and three replicates of dre2-4). Color keys are on the right. (PDF) [file pgen.1008094.s006.pdf]

A

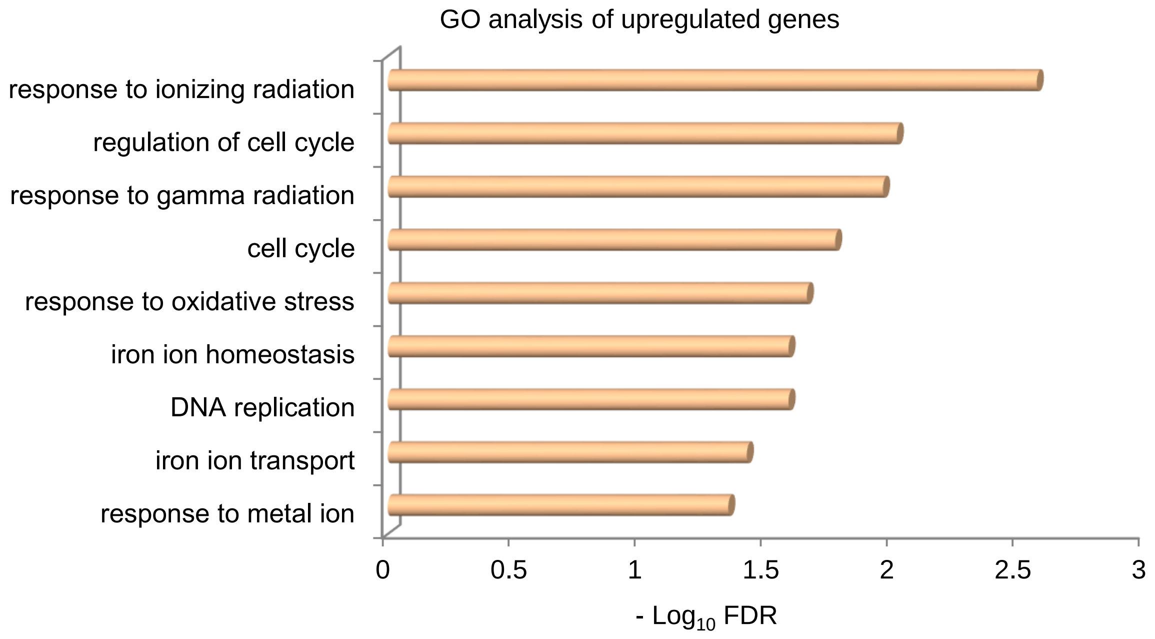

B

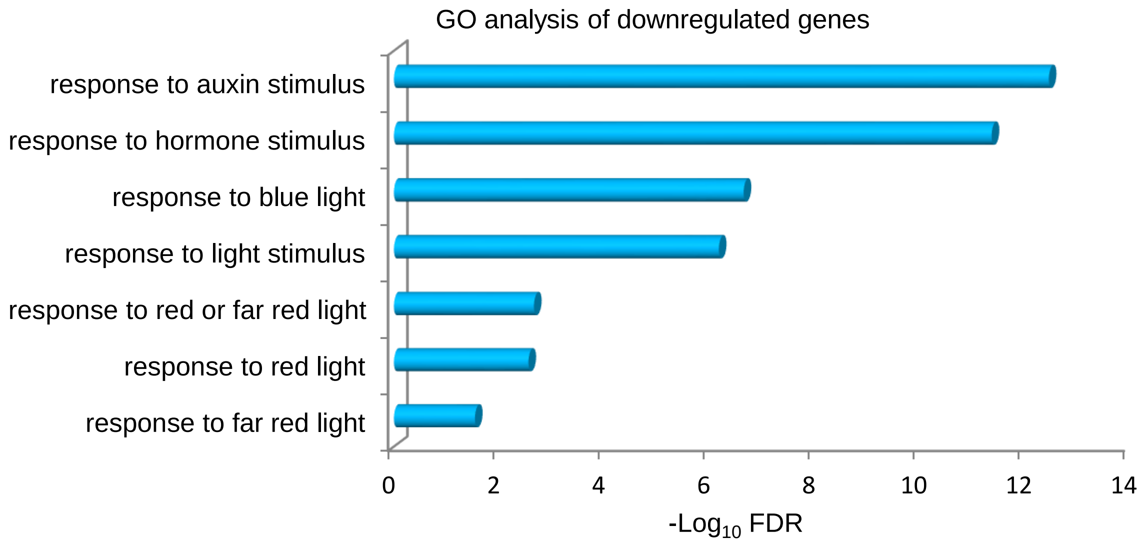

S6 Fig. Effects of *dre2-4* on RNA transcript levels as determined by RNA-seq.
(A-B) Gene Ontology analysis of significantly upregulated (A) and downregulated (B) genes in *dre2-4*. The lengths of bars represent statistical values of gene enrichment in the indicated biological processes. The biological processes listed are significantly (FDR<0.05) enriched. (C) Heatmap showing log_2 (FPKM+1) values of genes involved in DDR, cell cycle and DNA replication in the six samples (three replicates of Col-0 and three replicates of *dre2-4*). Color keys are on the right. (D) Heatmap showing log_2 (FPKM+1) values of genes involved in auxin response in the six samples (three replicates of Col-0 and three replicates of *dre2-4*). Color keys are on the right.

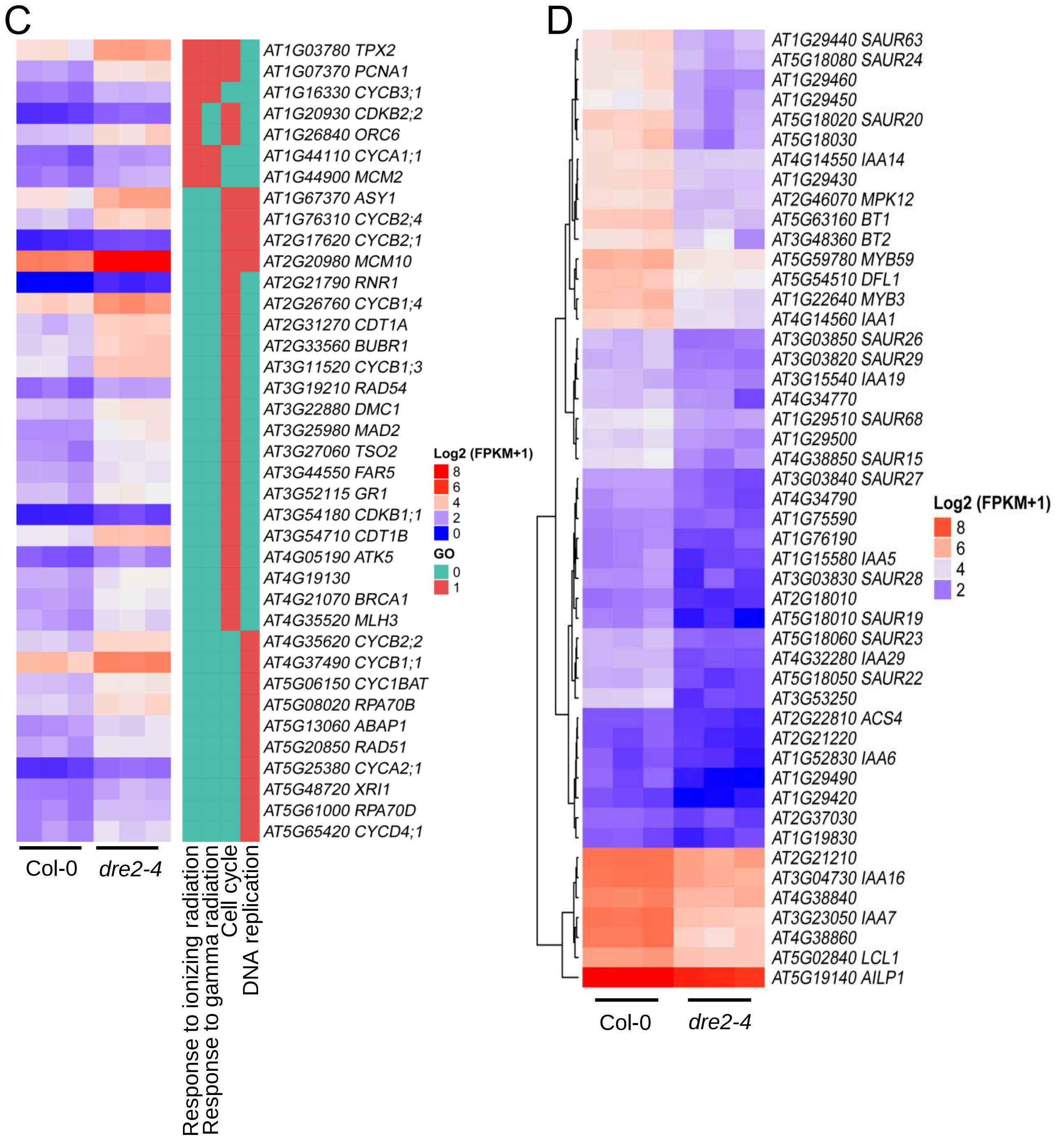
C
AT1G03780 TPX2
AT1G07370 PCNA1
AT1G16330 CYCB3;1
AT1G20930 CDKB2;2
AT1G26840 ORC6
AT1G44110 CYCA1;1
AT1G44900 MCM2
AT1G67370 ASY1
AT1G76310 CYCB2;4
AT2G17620 CYCB2;1
AT2G20980 MCM10
AT2G21790 RNR1
AT2G26760 CYCB1;4
AT2G31270 CDT1A
AT2G33560 BUBR1
AT3G11520 CYCB1;3
AT3G19210 RAD54
AT3G22880 DMC1
AT3G25980 MAD2
AT3G27060 TSO2
AT3G44550 FAR5
AT3G52115 GR1
AT3G54180 CDKB1;1
AT3G54710 CDT1B
AT4G05190 ATK5
AT4G19130
AT4G21070 BRCA1
AT4G35520 MLH3
AT4G35620 CYCB2;2
AT4G37490 CYCB1;1
AT5G06150 CYC1BAT
AT5G08020 RPA70B
AT5G13060 ABAP1
AT5G20850 RAD51
AT5G25380 CYCA2;1
AT5G48720 XRI1
AT5G61000 RPA70D
AT5G65420 CYCD4;1
Col-0
dre2-4
Response to ionizing radiation
Response to gamma radiation
Cell cycle
DNA replication
Log2 (FPKM+1)
8
6
4
2
0
GO
0
1
D
AT1G29440 SAUR63
AT5G18080 SAUR24
AT1G29460
AT1G29450
AT5G18020 SAUR20
AT5G18030
AT4G14550 IAA14
AT1G29430
AT2G46070 MPK12
AT5G63160 BT1
AT3G48360 BT2
AT5G59780 MYB59
AT5G54510 DFL1
AT1G22640 MYB3
AT4G14560 IAA1
AT3G03850 SAUR26
AT3G03820 SAUR29
AT3G15540 IAA19
AT4G34770
AT1G29510 SAUR68
AT1G29500
AT4G38850 SAUR15
AT3G03840 SAUR27
AT4G34790
AT1G75590
AT1G76190
AT1G15580 IAA5
AT3G03830 SAUR28
AT2G18010
AT5G18010 SAUR19
AT5G18060 SAUR23
AT4G32280 IAA29
AT5G18050 SAUR22
AT3G53250
AT2G22810 ACS4
AT2G21220
AT1G52830 IAA6
AT1G29490
AT1G29420
AT2G37030
AT1G19830
AT2G21210
AT3G04730 IAA16
AT4G38840
AT3G23050 IAA7
AT4G38860
AT5G02840 LCL1
AT5G19140 AILP1
Col-0
dre2-4
Log2 (FPKM+1)
8
6
4
2

S6 Fig
