# Supplementary material for: Canonical cytosolic iron-sulfur cluster assembly and non-canonical functions of DRE2 in Arabidopsis
Source: PLoS Genet. 2019 Apr 29;15(4):e1008094. doi: 10.1371/journal.pgen.1008094 (PMC6508740; doi:10.1371/journal.pgen.1008094)
Supplement: S7 Fig — (A) Primary root lengths of the indicated genotypes with or without 10 μM IAA treatment. (B) Relative primary root lengths of the indicated genotypes showing the inhibition of primary root growth by IAA. The ratios of primary root length after IAA treatment versus that without IAA treatment were calculated. (PDF) [file pgen.1008094.s007.pdf]

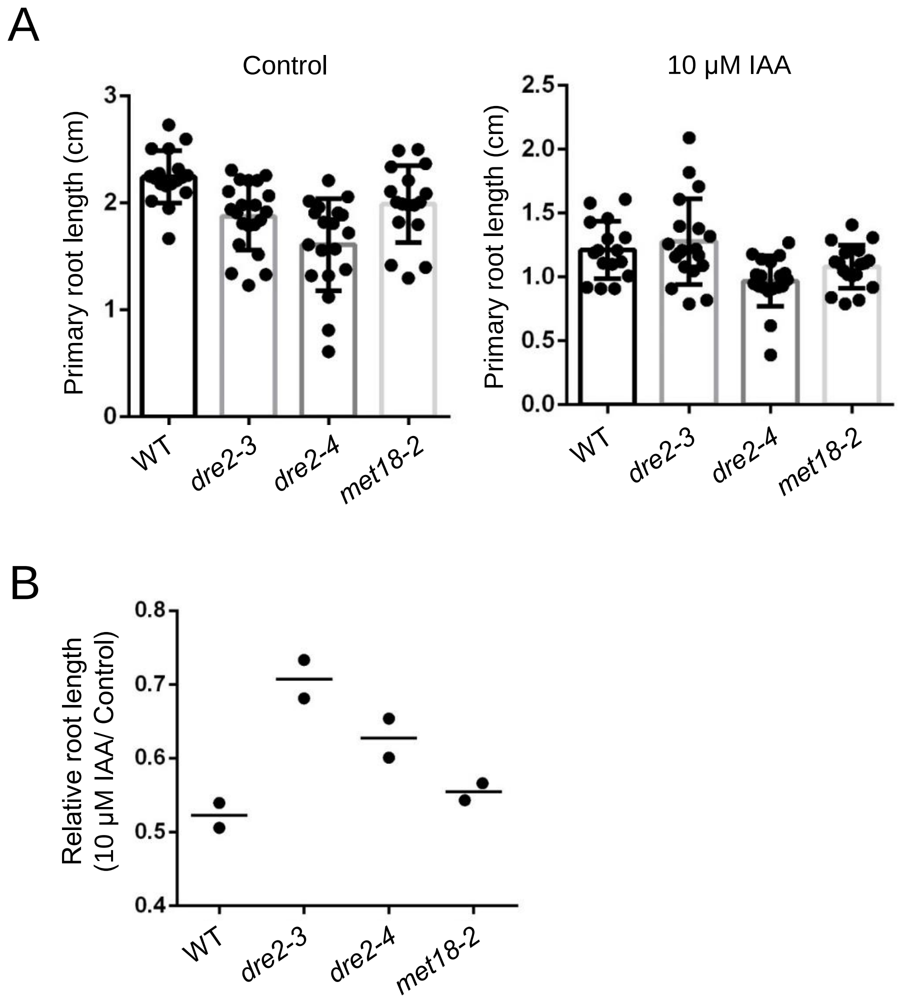

S7 Fig. Primary root length inhibition is alleviated in *dre2* mutants under IAA treatment. (A) Primary root lengths of the indicated genotypes with or without 10 μM IAA treatment. (B) Relative primary root lengths of the indicated genotypes showing the inhibition of primary root growth by IAA. The ratios of primary root length after IAA treatment versus that without IAA treatment were calculated.
