# Supplementary material for: Canonical cytosolic iron-sulfur cluster assembly and non-canonical functions of DRE2 in Arabidopsis
Source: PLoS Genet. 2019 Apr 29;15(4):e1008094. doi: 10.1371/journal.pgen.1008094 (PMC6508740; doi:10.1371/journal.pgen.1008094)
Supplement: S8 Fig — (A) DRE2-GFP expression in the differentiation zone of root without or with Leptomycin B treatment. (B) DRE2-GFP expression in the meristematic zone of root. (PDF) [file pgen.1008094.s008.pdf]

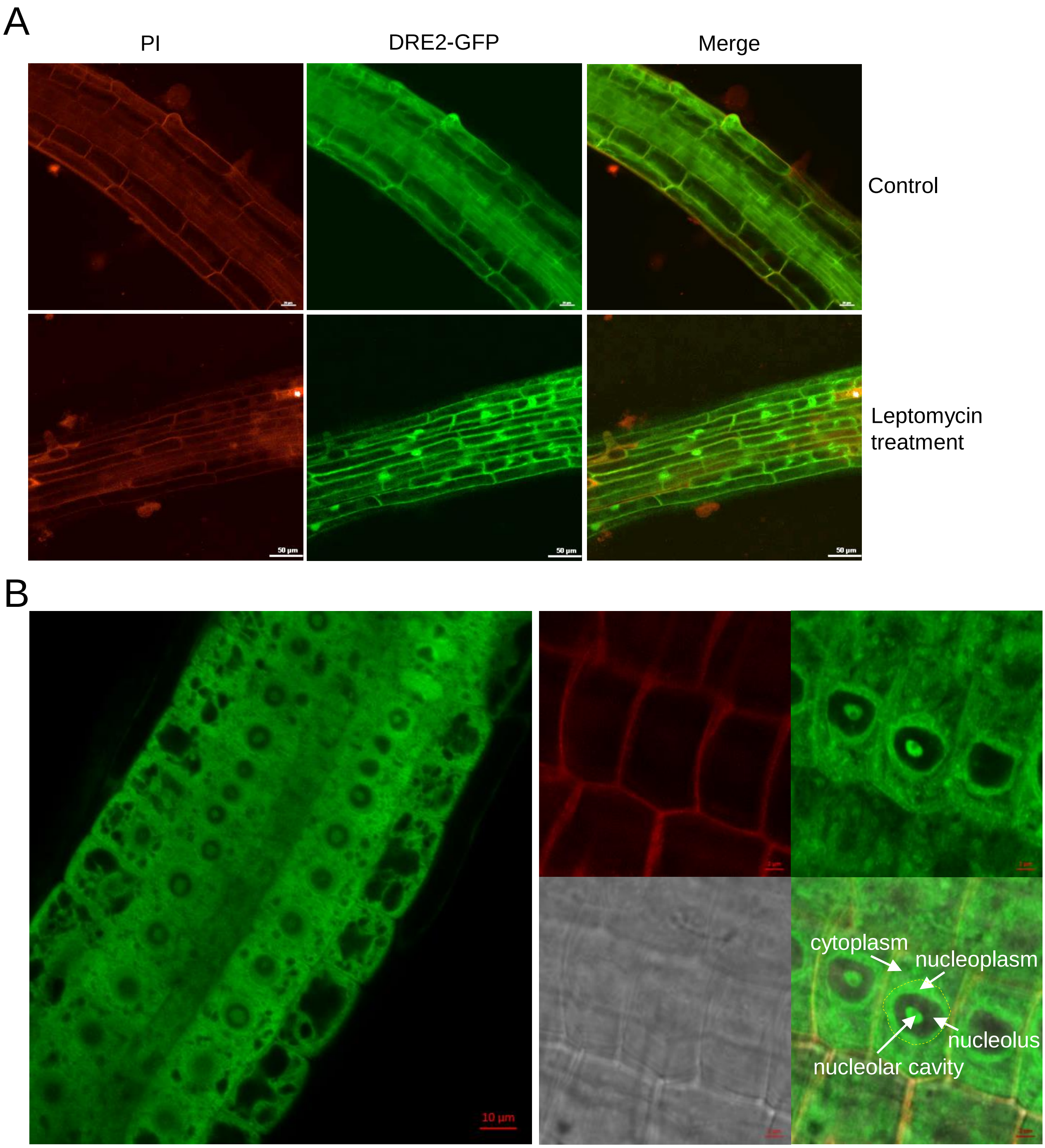

S8 Fig. Localization of DRE2-GFP.

(A) DRE2-GFP expression in the differentiation zone of root without or with Leptomycin B treatment. (B)) DRE2-GFP expression in the meristematic zone of root.
