# Supplementary material for: Canonical cytosolic iron-sulfur cluster assembly and non-canonical functions of DRE2 in Arabidopsis
Source: PLoS Genet. 2019 Apr 29;15(4):e1008094. doi: 10.1371/journal.pgen.1008094 (PMC6508740; doi:10.1371/journal.pgen.1008094)
Supplement: S9 Fig — (PDF) [file pgen.1008094.s009.pdf]

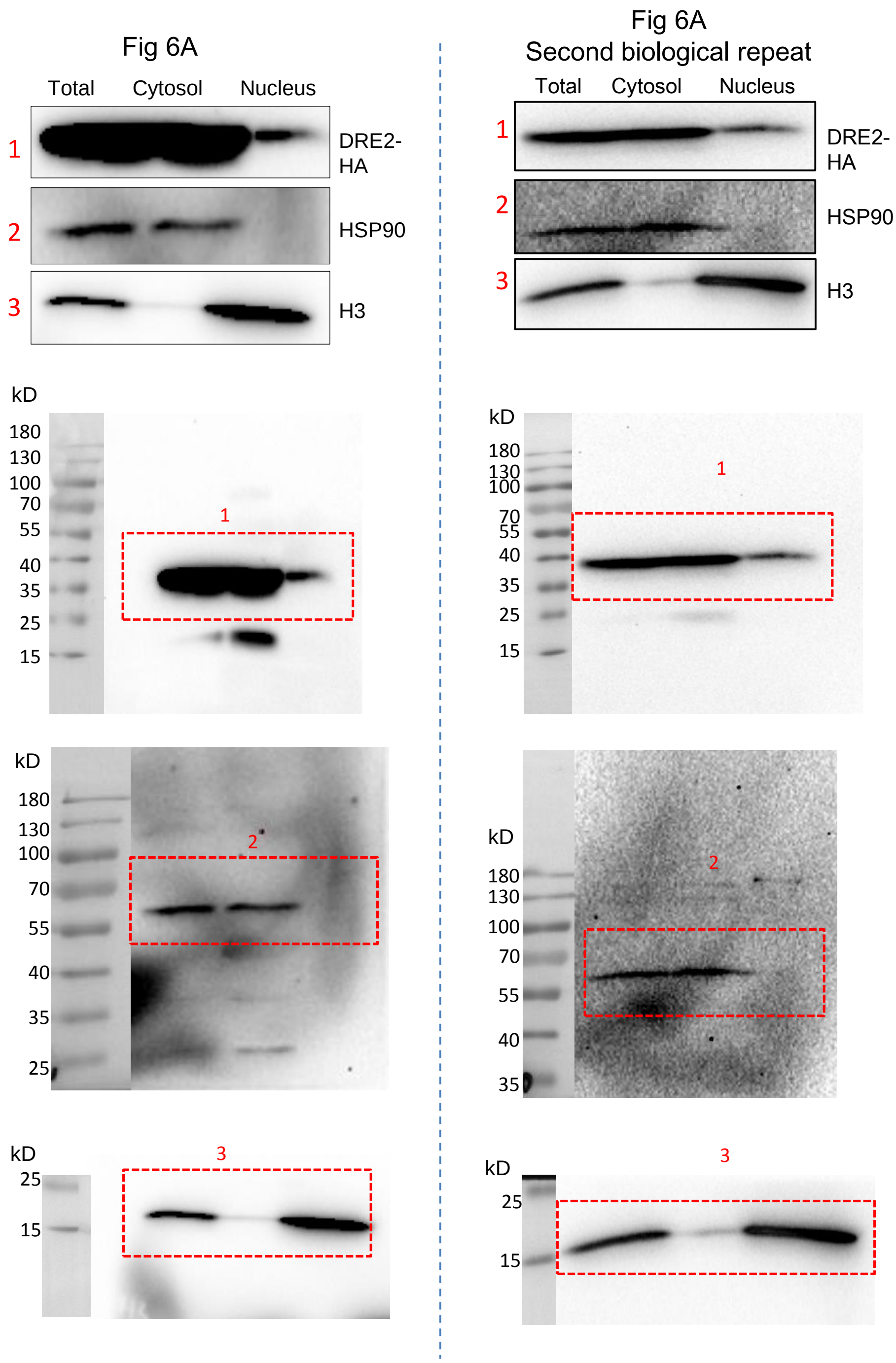

S9 Fig. Original Western blot data.

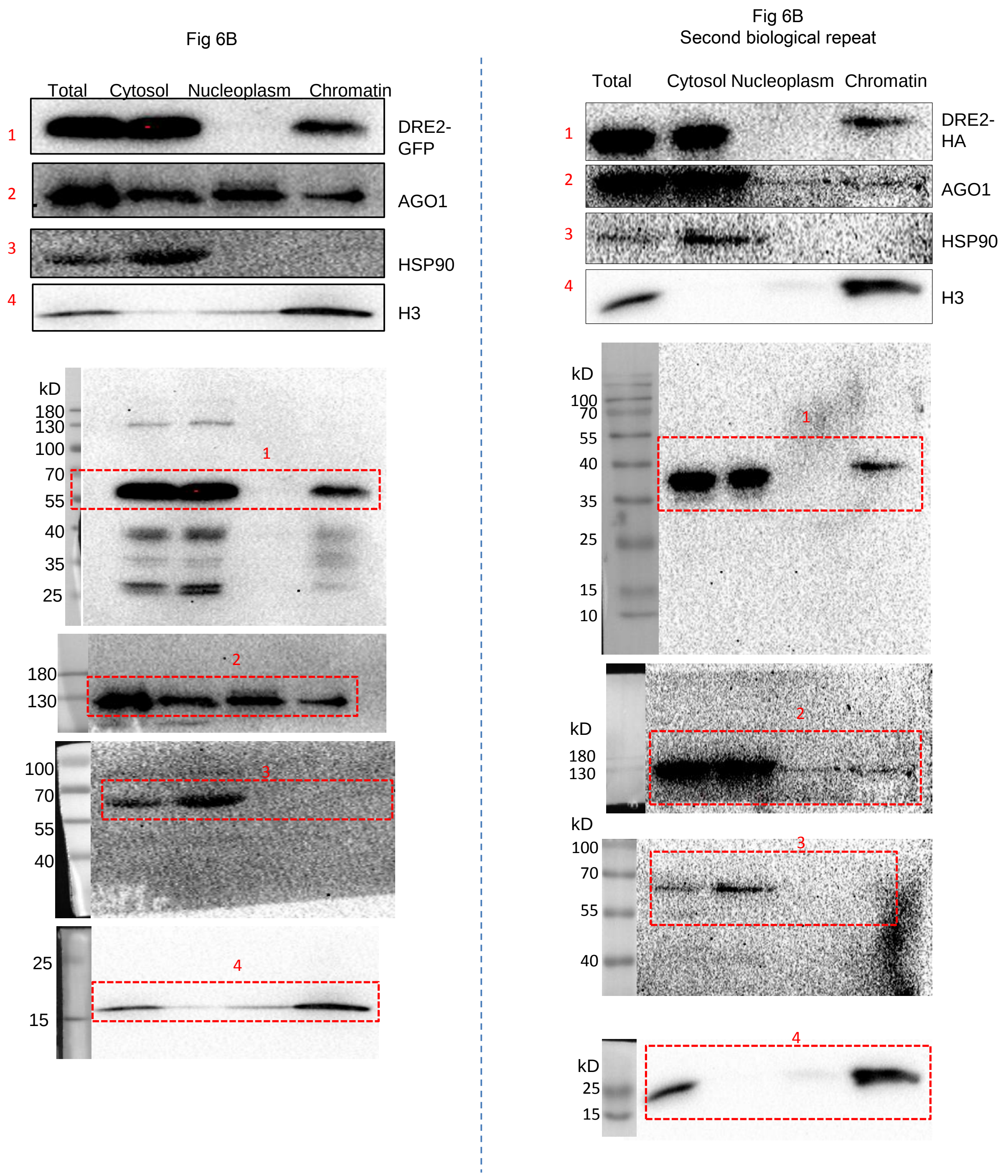

S9 Fig. Original Western blot data.
