# Supplementary material for: Canonical cytosolic iron-sulfur cluster assembly and non-canonical functions of DRE2 in Arabidopsis
Source: PLoS Genet. 2019 Apr 29;15(4):e1008094. doi: 10.1371/journal.pgen.1008094 (PMC6508740; doi:10.1371/journal.pgen.1008094)
Supplement: S1 Table — (PDF) [file pgen.1008094.s010.pdf]

Supplemental Table 1. Primers used in this study.

Primer Name	Sequence (5'-3')	Purpose
sgRNA1	TTTGGCTGTGACGGATGATG	CRISPR/Cas9-based *DRE2* gene editing
sgRNA2	AGGGTGGCAGACCCTTGTAA	
sgRNA3	TGTTGTCAAATGCAGATTA	
DRE2-Splicing-F	GAGAGTGACATTGGCTGGTT	Amplify splicing variants of *DRE2* in *dre2-4*
DRE2-Splicing-R	TGGCTTCATCAAGTCCTCCT	
DRE2-HA-F	ACGAATTCGAGCTCGGTACCGGCCTTCTACCACCTTA	Generate the *pDRE2::DRE2-HA* transgenic plant
DRE2-HA-R	ATGCCTGCAGGTCGACTATGTCAGCTTCAAGGAAGTTTT	
DRE2-GFP-F	CCAAGCTTGCATGCCTGCAGGGCCTTCTACCACCTTA	Generate the *pDRE2::DRE2-GFP* transgenic plant
DRE2-GFP-R	CCGGGGATCCTCTAGATATGTCAGCTTCAAGGAAGTTTT	
35S-DRE2-F	AGTCGGTACCATGGATTCGATGATGAATCAG	Complement *dre2-4*
35S-*DRE2*-R	AGTCGTCGACTTATATGTCAGCTTCAAGGAAG	
*ROS1*proBiF	TTTGATGATAAATTTATAAATAAAGTT	Bifulfite sequencing of *ROS1* promoter
*ROS1*proBiR	ATCACTAATACTTCATTTCTTCTCTT	
PARP1-F	TGCTCGCGCGAACTCACTTCT	RT-qPCR
PARP1-R	AGCCTCTCCACCAGAACGGCT	
PARP2-F	ATGGCGTTCTGCTCCTCTGC	
PARP2-R	GGTGCTGTTTTCCCCACACC	
PCNA1-F	GGGTTCTCACTCCAAGCTATG	
PCNA1-R	ATTCCTGTCGCATCTGTAGTG	
BRCA1-F	CCATGTATTTTGCAATGCGTG	
BRCA1-R	TGTGGAGCACCTCGAATCTCT	
GR1-F	GAAGGAGCAGACAAAGTGAG	
GR1-R	GGTGAGATGGAAGTGATAGG	
DMC1-F	AGGTATAAACGCAGGAGATGTG	
DMC1-R	CTTTGGCCTCAGATAAACCTTTG	
RAD51-F	CGAGGAAGGATCTCTTGCAG	
RAD51-R	GCACTAGTGAACCCCAGAGG	
RAD54-F	GATAACCCTGAGTGCGAAGAC	
RAD54-R	CAACTAGATTGCCCCTCAGAAG	
TSO2-F	TCGCTTGTCTACTCTACACG	

TSO2-R	CCGCGTCGCAGACGATTGA	
BARD1-F	TGCCCTCTATGCTTAAAATTGC	
BARD1-R	AGGACATCCCGATTCAACTTG	
Cyclin-B1-1-F	CAGACCATGCATACAGTCAC	
Cyclin-B1-1-R	TCCTAACTCCTAAGCAGATTC	
Cyclin-B1-2-F	CTGCTTGGACTGATACATTGC	
Cyclin-B1-2-R	CCATTCTCTGCCTTCGAGTAC	
Cyclin-B2-4-F	AATGAGTGTCGTAGCTGTGTC	
Cyclin-B2-4-R	ATTGTCAGGTAAAGCGTCTCC	
Cyclin-A1-1-F	CGATGGAGTTGAGAGGATGTG	
Cyclin-A1-1-R	CTCTTGTGGGATTACGGATGG	
Cyclin-A2-4-F	TCTCAAAGCATCTGTCCACG	
Cyclin-A2-4-R	GCTCTCCAGAACTGAATACCG	
ROS1-F	AAGGACCAACTTGTTGCGAC	
ROS1-R	AGGACTCTATTAGCACTGAGC	
AE7-F	TGAGAACCCTATCATTTACCCAAAG	
AE7-R	TGGTTCTGGTGTAAGCTCATC	
NAR1-F	AGTACCAAATCTCTACTGCAGC	
NAR1-R	GTCCGTTTCTCCACAGTCG	
NBP35-F	AAGGGCAAGAGATTCACCAG	
NBP35-R	TGAAACCTATGGACATGACACC	
DRE2-F	ATTTCTCCCGTTTCACCCTC	
DRE2-R	CTGTGGAGGCGGTATTAGC	
MET18-F	TCATTCGAGCGGCATACTG	
MET18-R	AGGGTGCTCTATGGTGAAAAG	
TAH18-F	GTTGTATGCCTCTCAGACCG	
TAH18-R	GAGGTAAGGAACTCGTGTCG	
CIA1-F	TCAGAAACTAGAAGGCCACAC	
CIA1-R	AGAGCTTTGTTCCCAGATTCG	
SAUR16-F	CTCCCTCTTGACGTACCAAAG	
SAUR16-R	TCAGGAATTCAGGATGAGTCAAG	
IAA14-F	GCTGGTGTACATCTTGAGGTC	
IAA14-R	CAGAGGAGGCAATGAGTAGTG	

PIN7-F	ATGCTCCATTCAAGACTACCG	
PIN7-R	TCCTCAAACAATCCTTACGCA	
PIN4-F	CTCGTCTTACTTGCTCTATGGG	
PIN4-R	AAGAGTGTTTGGGAGAGTGC	
DRE2-AD-F	AGTCCATATGATGGATTCGATGATGAATCAG	Generate the GAL4AD-fused wide-type and mutant DRE2 protein and generate DRE2$^{\Delta 75}$-6His recombinant protein
DRE2-AD-R	AGTCGGATCCTTATATGTCAGCTTCAAGGAAG	
TAH18-BD-F	AGCTCATATGATGGGAGAAAAACAAAGGAAGCT	Yeast-two-hybrid
TAH18-BD-R	AGCTGGATCCTTAAGACCAAGCTTCAACATTGT	
NBP35-BD-F	AGCTGAATTCATGGAGAACGGAGACATTCC	
NBP35-BD-R	AGCTGTCGACTCACTCGGTCATCACTGTTG	
GRXS17-BD-F	GGAGGACCTGCATATGATGAGCGGTACGGTGAAGGA	
GRXS17-BD-R	GGATCCCCGGGAATTCTTACTCGGATAGAGTTGCTTTGAGATC	
DRE2-pET28a-F	AGCTGGATCCATGGATTCGATGATGAATCAGA	Generate DRE2-6His recombinant protein
DRE2-pET28a-R	AGCTGTCGACTATGTCAGCTTCAAGGAAG	
IAA14-ChIP-F	CCTTCCGAAGATAAGGAGCTC	ChIP-qPCR
IAA14-ChIP-R	CAGAGGAGGCAATGAGTAGTG	
SAUR16-ChIP-F	TCTTGACTCATCCTGAATTCCTG	
SAUR16-ChIP-R	CAAGGGATGGTGAGTCCTC	
PIN4-ChIP-F	GTGGAGATGAAGTGGAAGGAG	
PIN4-ChIP-R	CTATGATTCTCGCCTACGGATC	
PIN7-ChIP-F	ACACGTAAATGCCGGTCCAAA	
PIN7-ChIP-R	TGGGCCAAATGCACACTTGTAT	
